# Data-driven exploration of electronic nose technology to differentiate bacteria in blood cultures under biofilm-promoting conditions

**DOI:** 10.1038/s41598-026-62071-8

**Published:** 2026-07-10

**Authors:** Julius Wörner, Nicole van Leuven, Jonas Eimler, Ulrich Odefey, Dirk P. Bockmühl, Miriam Pein-Hackelbusch

**Affiliations:** 1 Institute for Life Science Technologies (ILT.NRW), OWL University of Applied Sciences and Arts, 32657 Lemgo, Germany; 2 Faculty of Life Sciences, Rhine-Waal University of Applied Sciences, 47533 Kleve, Germany

**Keywords:** Biological techniques, Computational biology and bioinformatics, Microbiology

## Abstract

Biofilms are a major cause of delayed wound healing, yet current biofilm identification methods are limited by invasiveness, processing times, or specificity. This study investigates the potential of metal-oxide electronic noses for identifying bacterial cultures of *Staphylococcus aureus*,* Pseudomonas aeruginosa*,* Enterococcus faecium*, and *Staphylococcus epidermidis* grown in blood-based growth medium. We conducted in-vitro experiments to capture volatilome signatures from cultures grown under biofilm-promoting conditions and analyzed data using an interpretable machine learning workflow to disentangle algorithmic limitations from biological variability. This workflow incorporated feature extraction and selection, correlation-based clustering, and Shapley analysis. Six classification models were evaluated using cross-validation. Considering all five classes, classification accuracy reached at most 55.6%, which Shapley-based interpretation attributed mainly to biological factors: *E. faecium* and *S. aureus* exhibited high signal similarity to control samples and strong inter-day variability. Accuracy increased to 100.0% for species with distinct volatile signatures, and dimensionality reduction resulted in a model using two constructed features. These findings demonstrate that classification performance in biological sensing cannot be explained solely by algorithmic factors. Interpretable machine learning workflows help for distinguishing biological sources of complexity from algorithmic ones. We provide a proof-of-principle for electronic nose-based identification of bacterial growth under biofilm-promoting conditions.

**Supplementary Information:**

The online version contains supplementary material available at 10.1038/s41598-026-62071-8.

## Introduction

Bacteria can adhere to surfaces and form biofilms, structured microbial communities embedded in a self-produced extracellular polymeric matrix, which confer increased resistance to environmental stressors, host defense mechanisms and antimicrobial agents^1–5^. This phenomenon poses significant challenges to industries and particularly healthcare, where biofilms are frequently identified in sink drains, but also in infected wounds and implant sites^6,7^. Biofilms are present in up to 60% of chronic wounds and significantly impair wound healing, contributing to substantial clinical and economic burdens^8–12^. In addition to conventional methods for detecting biofilms, including imaging, molecular methods, culturing and biofilm blotting, several emerging approaches have been proposed. However, each suffers from limitations, such as long processing times, high costs, complexity, sample destruction, or the inability to distinguish between planktonic and biofilm state^9,10,13–17^. The need for biopsies additionally increases the risk for false negatives due to the heterogeneous distribution of the biofilms aggregates in the wound^10^. Among these, non-invasive or minimally invasive optical techniques, such as fluorescence imaging, Raman spectroscopy, and optical coherence tomography (OCT), have gained increasing attention^9,18–21^. Fluorescence imaging systems, such as the commercially available MolecuLight iX, exploit bacterial endogenous fluorescence and have been shown to significantly improve the detection of clinically relevant bacterial loads (> 10^4^ CFU/g tissue). Clinical studies report sensitivities of approximately 84% compared to 45% based on clinical symptoms alone, with a specificity of around 27%, while enabling real-time, non-contact assessment. Further, it offers very high practicability. However, limitations include high acquisition costs, learning time for image interpretation, restricted detection to fluorescent species, no value for bacterial count, no classification of the specific bacterial species present, and interference from host-derived autofluorescence or ambient light^9,22^. Raman spectroscopy offers high chemical specificity and has demonstrated the ability to differentiate bacterial species even within biofilm structures. It is a non-contact technique with rapid acquisition times, but its clinical translation is limited by strong signal attenuation from the extracellular polymeric substance matrix, sensitivity to fluorescence background, limited spatial coverage, and high system costs^20^. OCT provides structural, depth-resolved imaging of biofilms and has been successfully applied to visualize biofilms, as demonstrated in clinical and experimental studies^18,21,23^. It enables real-time 3D imaging and quantitative assessment. However, its implementation is hindered by high equipment costs^24^. Overall, while these emerging methods offer substantial advantages in terms of speed, real-time capability, and reduced invasiveness compared to conventional techniques, none of them currently provides complete overage of all clinically relevant biofilms or fully overcome challenges related to specificity, costs, and practical implementation in existing clinical workflows. Another promising new method of detecting biofilms is the analysis of Volatile Organic Compounds (VOCs) emitted from the bacteria. Species-specific volatile compounds have been linked to biofilm physiology and viability, and their concentrations change in response to biofilm development, disruption, and antibiotic treatment, highlighting their potential as non-invasive biomarkers^3,25^. Further, the metabolic output of the bacteria is heavily influenced by host interactions, which also may alter the set of produced metabolites^26^. The most critical measures for future diagnostic tests include the ability to discriminate between planktonic and biofilm states, the identification of specific bacterial species present, the determination of the dominant species within a biofilm, and the localization of the biofilm within wounds^10^. Recent studies have begun to explore whether VOC-based detection and identification can meet these requirements. Ashrafi et al.^3^ showed that VOC profiles from biofilm-forming methicillin-sensitive *S. aureus*,* P. aeruginosa*,* and S. pyogenes* can be differentiated using gas chromatography-mass spectrometry (GC-MS). Specific VOCs were uniquely associated with each species and reflected biofilm development. Moreover, antibiotic treatment significantly reduced key VOCs. Besides GC-MS, electronic noses (e-noses) can also measure volatiles. These systems enable the sensing and detection of gaseous mixtures including VOCs^27–29^. Through significant technological advancement over the last decades, e-noses have been successfully explored in many areas and now hold promise for addressing challenges in biofilm detection^27,29,30^. Thaler et al.^31^ used an e-nose combined with logistic regression to distinguish biofilm- from non-biofilm-producing strains of *P. aeruginosa* and *S. aureus*, achieving accuracies of 72 to 100%. These studies demonstrate that biofilm growth produces distinct volatile patterns suitable for non-invasive detection.

Despite these promising results, a critical gap remains in translating findings from standard nutrient broths to physiologically relevant environments. Standard media fail to reflect the complex chemical matrix of a bleeding wound, which could potentially interfere with VOC detection. Therefore, we evaluate the discriminatory power of e-nose technology for bacteria cultivated in a biofilm-promoting blood-based medium. We examined four distinct pathogens to determine if their established VOC profiles^32–37^ remain distinguishable within this more complex biological matrix. However, the successful translation of e-nose technology into (clinical) practice relies not only on experimental design but also on robust data analysis^38^. But analyzing data from metal-oxide gas sensor arrays presents challenges such as high-dimensionality and the risk of overfitting, especially with limited biological samples^39–41^. These challenges are compounded by biological variability, such as biofilm formation and blood contamination, as well as technical constraints including signal drift and information loss. Therefore, reliable analysis requires both appropriate preprocessing and interpretable models that enable meaningful biological contextualization.

To address these biological and algorithmic challenges, we applied a comprehensive data processing workflow that captures static and dynamic sensor features, followed by feature selection and correlation-based clustering for dimensionality reduction. We evaluated different classification models with varying complexity and decision boundaries using a leave-one-day-out cross-validation. We employed Shapley values to provide model interpretability, revealing which response characteristic of the sensors drive biofilm differentiation. Shapley values quantify the impact of each feature by considering its contribution across all possible combinations of features, providing a measure of feature importance^42^. This is the first study to assess the VOC-based discriminability of bacterial cultures of these four pathogen species grown under biofilm-promoting conditions in blood-based growth medium using an e-nose. Further, measurements were taken after less than 20 h of incubation to capture early bacterial growth. By conducting three independent in vitro measurements using fresh cultures on three different days, we address the influence of background volatiles from blood components. The presented study was designed as an explorative proof-of-principle to investigate whether VOC profiles enable discrimination between clinically relevant biofilm-forming bacterial species under conditions that promote biofilm development. A modified Lubbock chronic wound biofilm model was employed, providing a blood-based environment with a solid-liquid interface and shear forces known to facilitate bacterial attachment and biofilm formation^43–45^. All experiments were conducted under identical, established biofilm-promoting conditions to provide a reproducible basis for these initial investigations. Through combining this experimental approach with the described data analysis, we demonstrate the theoretical feasibility of e-nose technology for bacterial differentiation under more complex conditions, aiming to pave the way for fast, point-of-care diagnosis of infected wounds in clinical settings.

## Materials and methods

### Preparation of biofilm samples

Strains of *Pseudomonas aeruginosa* (DSM 939), *Staphylococcus aureus* (DSM 799), *Enterococcus faecium* (DSM 2146) and *Staphylococcus epidermidis* (DSM 1798) were used for analysis. Biofilms were cultivated using a modified Lubbock chronic wound pathogenic biofilm model originally described by Sun et al., in which bacterial biofilms are formed on polymeric pipette tips^43^. The model provides a solid-liquid interface that promotes bacterial attachment while exposing the tips to liquid shear forces during incubation. We used a blood-based medium consisting of standard Columbia Nalidixic Acid agar without selective supplements to allow the cultivation of all four bacterial species under clinically relevant conditions. Polypropylene pipette tips were selected as the adhesion surface as polypropylene is a biologically inert polymer widely used in medicine, including sutures, surgical meshes, and oxygenator membranes, thereby providing a clinically relevant substrate for bacterial attachment^46^.

Liquid cultures of each species were prepared from agar plates and incubated in Tryptic Soy Broth (TSB). The cell density of the resulting suspension was determined by measuring the optical density at 600 nm (OD600) and adjusting to 0.5 ± 0.04. The bacterial cultures were then used to induce bacterial biofilm formation. The culture medium was based on a Columbia Nalidixic Acid agar. To prepare the medium, following concentrations of ingredients were used: 12 g/L peptone from casein (Merck KgaA, Darmstadt, Germany, Cat. No. 1.11931.1000), 5 g/L peptone from gelatin (Sigma-Aldrich, Merck KgaA, Darmstadt, Germany, Cat. No. 70176-500G), 3 g/L yeast extract (Carl Roth GmbH + Co. KG, Karlsruhe, Germany, Cat. No. 2363.2), 3 g/L meat extract paste (Carl Roth GmbH + Co. KG, Karlsruhe, Germany, Cat. No. 5770.1), 1 g/L starch from corn (Carl Roth GmbH + Co. KG, Karlsruhe, Germany, Cat. No. 9444.1) and 5 g/L NaCl. All ingredients were filled up and diluted in distilled water. After autoclaving, the medium was supplemented with 10% (v/v) defibrinated sheep blood (Thermo Fisher Scientific Inc., Waltham, MA, USA) and filled in autoclaved vials in 10 mL aliquots for further preparation of the inoculated samples.

10 µL of each bacterial suspension (OD600 of 0.5 ± 0.04) were taken up with an Eppendorf micropipette (Eppendorf Research plus 10–100 µL, Eppendorf SE, Hamburg, Germany) and the filled tip was directly added to the prepared vials with culture medium (20 µL pipette tips, Sarstedt AG & Co. KG, Nümbrecht, Germany). For the control samples, a pipette tip filled only with pure culture medium was used.

The vials were cultivated in a shaking incubator (IKA KS 4000 i control, IKA-Werke GmbH & Co. KG, Staufen, Germany) at 37 °C and 125 rpm overnight for about 17 h, aiming for bacterial biofilm formations on the pipette tip surfaces. For each species, three biological replicates were cultivated and analyzed each day.

**Fig. 1:**
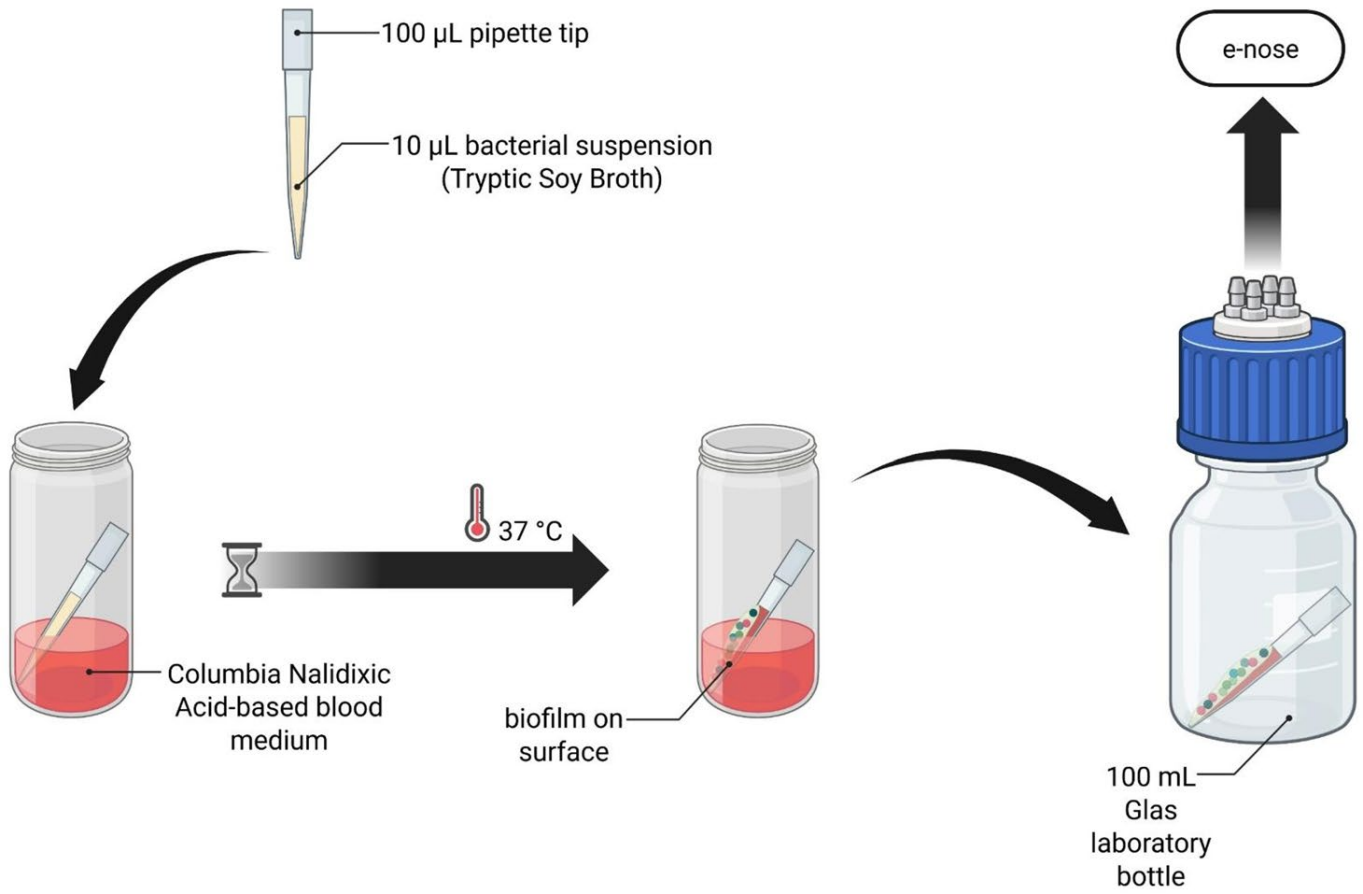
Schematic of the experimental procedure of biofilm cultivation in blood-based growth medium. Pipette tips with bacterial suspension were cultivated overnight at 37 °C to induce biofilm formation. Created in BioRender. Pein-Hackelbusch, M. (2026) https://BioRender.com/z9zzfre.

At the beginning of each of the three days, all vials were taken out of the incubator. The pipette tips were transferred into 100 mL Schott glass bottles along with the liquid remaining inside the tips. Three bottles were prepared for further measurement, while the remaining tips were kept in the blood medium until further preparation to prevent drying. The whole experimental procedure is summarized in Fig. 1.

### Experimental setup and measurement of samples

#### Electronic nose and experimental setup

Measurements were performed using a commercially available metal-oxide-based e-nose system (Smelldect GmbH, Deckenpfronn, Germany). The sensor array features 62 SnO_2_ nanowires excited by ultraviolet light, allowing operation at lower temperatures than usually for heated metal-oxide gas sensors. A detailed description of the sensor system, the measurement cycle and the general gas flow setup can be found in our previous work^47^. The main working principle of these nanowires is based on the interaction between the metal oxide surface of the sensors and the gas molecules. Chemical reactions due to the adsorption of target gas molecules change the resistance of the sensors^48^. Compressed air as carrier gas was used to reduce ambient air influences.

The experimental setup is shown in Supplementary Figure S1. The measurement cycle followed the previously described protocol^47^, consisting of sensor flushing, liquid-gas equilibrium establishment, and sample measurement. Flushing the sample bottle with clean air prior each measurement reduced the influence of volatiles from ambient air. The overall carrier gas flow rate was set to 100 sccm. By passing 60% of the flow through deionized water (mass flow controllers: Smart Controller GSC, red-y smart series, Voegtlin Instruments GmbH, Muttenz, Switzerland), the relative humidity in the sensor chamber was adjusted to approximately 75%. The measurements were controlled using the manufacturer’s software (Kamina Observer Version 2.0, Karlsruher Institute of Technology (KIT), Karlsruhe, Germany, 2013 − 202).

#### Measurement procedure

Measurements were started 15–30 min after e-nose start-up to avoid thermal effects from the initial warm-up phase, characterized by a rapid temperature increase in the closed sensor chamber due to heat dissipation from the UV lamp. Stabilization was confirmed by a reduced temperature gradient, with maximum changes of approximately 0.2 °C per minute, resulting in temperature-induced resistance variations within the sensor noise.

Overnight measurements using an empty Schott glass bottle under identical conditions served to verify the stability of the compressed air supply and to purge the whole system with clean air. All measurements were carried out under a fume hood for stable environmental conditions.

The duration of a whole measurement cycle for a single sample was set to 20 min. This duration covers sensor flushing, liquid-gas equilibrium establishment, sample measurement, and sensor recovery, each of which lasting 5 min.

Sample measurements were conducted on three consecutive days. For each bacterial species, three individual samples were prepared each day. In addition to these measurements, repeated measurements were performed after intermediate waiting periods to allow partial drying of the biofilms, thereby increasing variability in the dataset. Due to differences in the number of error-free measurements and repeated measurements, the total number of samples was as follows: 12x control, 12x *E. faecium*, 11x *P. aeruginosa*, 11x *S. aureus*,* and* 10x *S. epidermidis.* The order of sample measurements was randomized to minimize sensor drift influences.

### Data processing

#### Calculation of features

For each sample measurement, the sensors provide a time series of resistance values across the different phases, rather than a single interpretable value per sample^47^. A visualization of a single resistance curve is given in Fig. S2 in the Supplementary Materials. Each sensor thus produces high-dimensional response curves, in which temporally close resistance values are highly correlated. There are two main approaches to use these response curves for further analysis: (1) using the curves directly as input to models such as neural networks, or (2) extracting features from these curves to describe the sensor behavior and response in a lower-dimensional space, ideally without losing important information^27,38^. Especially when the dataset is small, dimensionality reduction can be a crucial preprocessing step to improve model performance by building more effective feature spaces^38,39^.

Numerous features that can be derived from gas sensor responses in e-nose systems have been reported in literature^27^. Because there is no universally valid recommendation for good features, and the optimal feature set depends strongly on the specific sensor type and, above all, on the particular application, we extracted several different features in this study. This resulted in a high-dimensional dataset comprising a total of 1,618 features. Specifically, 26 features were extracted from each gas sensor response, while three additional features were computed for the temperature and humidity sensor (i.e., mean, absolute difference, and relative difference). A detailed description of all features can be found in the Supplementary Materials (Table S1). Our feature set included both features capturing the dynamic behavior of the sensor signals, which can add important information, as well as static features^27^. Steady-state features quantified absolute and relative response magnitudes, e.g. mean values, final responses and fractional differences. Further, these features quantified recovery levels to assess saturation. Dynamic features comprised rate-of-change metrics, like the initial slope and derivatives, but also temporal milestones (e.g., T50 recovery time) and integrals. We further added the skewness of the distribution as a transient feature to determine signal stability, reaction speeds, and desorption kinetics. All features were extracted from the raw sensor data stored in a .csv file. For calculating the features, Jupyter Notebook v6.4.8 with Python 3.9.12 was used. The resulting dataset with 1,618 features and 56 samples was then used for further pre-processing steps.

#### Normalization

To normalize the data before training the classifiers, we evaluated two different normalization methods for each classifier to ensure compatibility and optimal model performance. The first method applied standard scaling, where each new value is calculated as in Eq. (1):

1 $$\:{x}_{new}=\:\frac{x-\stackrel{-}{x}}{s}$$

using the mean $\:\stackrel{-}{x}$ and the standard deviation s of the feature^39^. This results in a mean of 0 and standard deviation of 1 for all features. The second method considered is a more robust scaling approach, which is less sensitive to outliers, as it uses the median $\:{x}_{median}$ and interquartile range (IQR) instead of the mean and standard deviation. These scaled values were calculated according to Eq. (2):

2 $$\:{x}_{new}=\:\frac{x-{x}_{median}}{IQR}$$

where the IQR is defined as the range between the 1st quartile and 3rd quartile^49^ .

#### Feature selection

To reduce the initial number of features, we first identified the most informative features. We employed feature ranking based on F-statistics derived from ANOVA. The F-test was applied pairwise to all combinations of classes for each individual feature. For each class pair, the 20 features with the highest F-scores were selected. The number of selected features per class pair was determined empirically based on preliminary experiments.

The F-ratio is defined as in Eq. (3):

3 $$\:F=\:\frac{variability\:between\:groups}{variability\:within\:groups}$$

The F-ratios were computed using the *f_classif* score-function from scikit-learn, which implements the classical ANOVA F-test.

The final feature set was constructed by taking the union of all features selected across the pairwise class comparisons. This univariate feature selection approach evaluates each feature independently and therefore does not capture interactions or correlations between features. To address this, a correlation-based dimensionality reduction technique was applied to further reduce dimensionality.

#### Clustering with correlations

As an optional preprocessing step, we evaluated correlation-based clustering to reduce feature redundancy. This is particularly useful when variables are highly correlated, as is often the case for gas sensor signals. In such situations, a lower-dimensional representation could retain most of the relevant information. This reduction was achieved by clustering features according to their correlations to reduce redundancy arising from these strong inter-feature correlations. The goal of this method is to group highly correlated features and represent them with fewer variables, thereby reducing dimensionality while preserving informative structures. We performed agglomerative hierarchical clustering based on distances calculated with pairwise correlations. Agglomerative hierarchical clustering starts with each feature forming its own cluster and iteratively merges the two most similar clusters until larger clusters are formed^50^.

To apply this method, we first defined a distance measure between features based on their correlation. The distance matrix *D* was defined by Eq. (4):

4 $$D_{{ij}} = \left( {\begin{array}{*{20}c} 0 & \cdots & {d_{{1n}} } \\ \vdots & \ddots & \vdots \\ {d_{{n1}} } & \cdots & 0 \\ \end{array} } \right)$$

where the distance between feature *i* and feature *j* was defined as in Eq. (5):

5 $$\:{d}_{ij}=1-\left|corr\left({f}_{i}{,f}_{j}\right)\right|\:\:\:\:\:\:\:,\:i,j=1,\dots\:,n$$

with *n* being the number of features. Pairwise correlations were calculated using the Pearson correlation coefficient. This definition ensures that strongly correlated features (either positively or negatively) have small distances, while uncorrelated features have a distance close to 1. Taking the absolute value of the correlation treats positive and negative correlations equally, as both indicate redundancy.

The optimal clustering threshold (i.e., the maximum distance up to which clusters are still merged) was determined using grid search. For merging clusters, we used maximum linkage, where the distance between two clusters is defined as the largest distance between any pair of features from the two clusters. Finally, to obtain a reduced feature representation, we evaluated four approaches for reducing each new cluster into a lower-dimensional form: (1) retaining only the one feature with the highest F-score within the cluster, (2) selecting the first feature in the cluster, (3) averaging all features within the cluster, and (4) applying linear discriminant analysis (LDA) to each cluster and transforming the clusters into new features by projecting the data onto the discriminant components.

This resulting reduced feature set was then used to train and evaluate different classification models. The method was performed during cross-validation.

### Classification

In this study we considered six commonly used classification algorithms: *Logistic Regression (Multinomial Logistic Regression)*,* Support Vector Machine (SVM)*,* Decision Trees and Random Forests*,* Gradient Boosting and k-Nearest-Neighbors (kNN).* The bacterial species were used as class labels, yielding a five-class classification problem as control samples were included. Model evaluation and generalization tests were performed using leave-one-day-out cross-validation. The total sample size per class ranged from 10 to 12 samples. In each fold, data from one whole day were held out as test set, resulting in approximately 3 to 4 samples per class in the test set and the remaining samples in the training set. Model performance was assessed using different complementary metrics, including accuracy, precision, recall, F1-score and specificity. In addition, we used a confusion matrix to visualize class-specific predictions and misclassifications. We implemented all models in Python using the scikit-learn library.

**Fig. 2:**
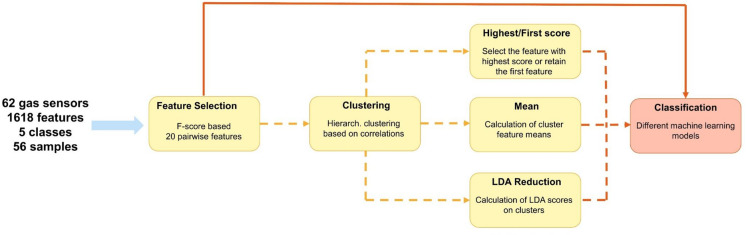
Overview of the data preprocessing workflow. Dashed lines indicate optional intermediate steps. The preprocessing includes feature selection followed by optional clustering, with various methods tested to summarize the feature values within a cluster.

The whole preprocessing workflow is summarized in Fig. 2. Dotted lines indicate optional steps, which were tested but not mandatorily used for all classification models.

### Feature analysis

SHAPLEY values were used in this study to interpret the contribution of individual features to the predictions of the model. In this study, the SHAPLEY values were computed using the SHAP Python package (version 0.49.1).

## Results and discussion

### Multiclass classification across all bacterial species

After approximately 17 h of incubation, biofilm formation was observed on pipette tips, and the structures were visually detectable across experimental conditions. In total, 56 samples were included in the analysis, bacterial cultures derived from the four pathogens, as well as control samples. Initial data-driven analysis was performed on the complete dataset including the sensor signals for all bacterial samples before conducting subgroup-specific analyses. We used cross-validation to evaluate the classification performance across measurement days. When considering the complete dataset, despite hyperparameter optimization, the maximum classification performance was below 60% across all models. Hierarchical correlation clustering did not consistently improve results, and the choice of normalization as well as the method used to aggregate cluster values varied across models. These observations highlight that optimal preprocessing strategies depend on both the classifier and the specific characteristics of the dataset. The final parameter sets and methods are provided in Supplementary Notes of the Supplementary Materials. The confusion matrix of the two best-performing models revealed frequent confusion between the species *E. faecium* with the control samples (see Supplementary Materials Table S2 and S3). This was likewise the case for the remaining classification models. To investigate whether feature stability, suboptimal classifier performance or biological reasons played a role in this effect, we analyzed the feature sets selected with the method described ‘Materials and Methods’ for each cross-validation training split.

Features were selected based on F-scores, prioritizing variables with stronger systematic effects and therefore with high inter-class differences and low within-class variability^51^. Feature selection varied across cross-validation folds, with only about 20% consistently selected. However, many fold-specific features were strongly correlated, indicating pronounced multicollinearity within the feature space. This suggests that different feature subsets reflect interchangeable representations rather than fundamentally distinct discriminative patterns, likely driven by small stochastic differences in the training data.

**Fig. 3:**
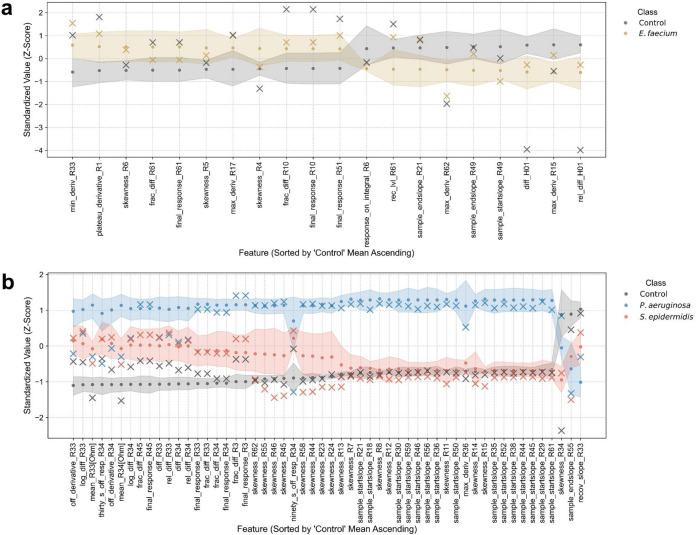
Line plot of the selected features with the highest scores. Points represent mean values over the training days, while crosses indicate mean values of the test day. Shaded areas represent the 99.5% confidence interval, calculated using the standard error of mean. The first two days were used as training set and the third day as test set (i.e., fold 1). For E. faecium and control samples (**a**) the confidence intervals show overlaps and the test samples lie outside the intervals of the respective class. For *P. aeruginosa*,* S. epidermidis* and control samples (**b**), many features exhibiting non-overlapping intervals.

As shown in Fig. 3a for the first fold (i.e., first and second day as training data), the substantial overlap of confidence intervals between control samples and *E. faecium* across the top-ranked features indicates limited discriminative power. Because the top 20 features were selected separately for each pairwise class comparison according to their F-scores, the number of displayed features differ between Fig. 3a and b. Figure 3a shows that the test samples deviate markedly from these intervals, pointing to variability effects that mask underlying separation. Together, these observations suggest that the underlying signals may already exhibit considerable similarity between these classes, potentially contributing to the limited classification performance. In contrast, Fig. 3b reveals minimal overlap of confidence intervals between control samples and *P. aeruginosa* for many features, indicating distinct volatile signatures detectable by the sensor system. This is consistent with studies describing a broader range of species-specific metabolites for *P. aeruginosa* compared to *S. aureus* or *E. faecalis*^26^. For *S. epidermidis*, some features also show separation from controls, although less consistently. Taken together, these findings provide initial evidence that *P. aeruginosa*, and to a lesser extent *S. epidermidis*, may be distinguishable even based on single features, whereas the pronounced overlap observed for *E. faecium* suggests that reduced separability may originate from similarities in the underlying signal space rather than solely from limitations of the applied models.

### Separating class overlaps from algorithmic bias

To further investigate the influence of *E. faecium* samples on the model performance, we removed this bacterial species from the dataset and created a new subset. With this reduced classification setting, the performance of the classifiers increased. These findings indicate that *E. faecium* is a critical species affecting classification performance, with all models consistently struggling to discriminate between the control samples and the *E. faecium* samples. This is further reflected in the recall values of the models for *E. faecium* and the control class. Models showing higher recall for *E. faecium*, such as kNN (0.67) simultaneously exhibited markedly reduced recall for the control class (0.08).

Literature indicates that *E. faecium*, compared to other microorganisms investigated in the respective studies, often produces fewer and more variable VOCs in lower quantity, influenced by strain differences and growth conditions, including nutrient composition^52^. For example, diacetyl, to which our sensors are very sensitive^53^, occurs in some conditions only at low levels in *E. faecium* profiles. In general, it has been demonstrated that VOC production varies considerably between strains, genomic variations and depends strongly on nutrient composition, with diacetyl being built in markedly different quantities under different conditions^26,54^. Consequently, key discriminatory volatiles may have been present only at low concentrations in our experimental setting, limiting their detectability by the sensor array. In contrast, *P. aeruginosa* generates a broader, more distinctive VOC spectrum, facilitating classification^26^. These factors likely explain the limited separability of *E. faecium* observed in our study, consistent with previous reports that only a small fraction of bacterial volatiles is uniquely produced by only one species^26^.

Examination of the confusion matrix also revealed that misclassifications predominantly involved confusion between *S. epidermidis* and *S. aureus*. As discussed later, this pattern reflects non-robust and variable sensor responses, which may have obscured subtle differences between these bacterial cultures and reduced classification stability. After excluding *E. faecium* and *S. aureus*, the classifiers were retrained on a reduced subset. A substantial improvement in accuracy and class assignment was observed, reaching a maximum accuracy of 100% (Tabl. 1). The confusion matrix of the kNN-classifier and Random Forest are given in Supplementary Materials Table S4 and S5. This pronounced performance gain on the reduced subset underscores that these two classes that were left out systematically challenged the models. As shown in the following, this is likely attributable to biological or sensor array constraints rather than algorithmic constraints.

**Table 1:** Class-wise recall for every classifier (computed on the aggregated confusion matrix), and mean cross-validation accuracies (%) averaged across folds, reported for the derived hyperparameter sets and evaluated using cross-validation. SVM = Support Vector Machine, kNN = k-Nearest-Neighbors, DT = Decision Tree, LR = Logistic Regression, GB = Gradient Boosting and RF = Random Forest.

	Class	SVM	kNN	DT	LR	GB	RF
5-class classification	*S. epidermidis*	30.0	30.0	20.0	30.0	30.0	10.0
	*P. aeruginosa*	100.0	100.0	100.0	100.0	100.0	100.0
	*E. faecium*	50.0	66.7	58.3	41.7	41.7	41.7
	*S. aureus*	45.5	54.5	36.4	45.5	63.6	54.5
	*Control*	25.0	8.3	41.7	41.7	58.3	50.0
	Mean CV Accuracy	50.4	52.2	52.0	52.2	**59.3**	
3-class classification	*S. epidermidis*	50.0	60.0	100.0	60.0	50.0	80.0
	*P. aeruginosa*	90.9	100.0	100.0	100.0	90.9	100.0
	*Control*	100.0	100.0	100.0	91.7	100.0	91.7
	Mean CV Accuracy	81.2	88.9	**100.0**	85.6	82.8	91.2

Classification performance varied substantially across folds. Except for SVM, all classifiers achieved 100% accuracy in at least one fold, indicating that good separation was possible in some training-test splits. However, kNN accuracy ranged from 66.7% (fold 3) to 100% (folds 1 and 2), illustrating the impact of sampling variability on performance estimates. It will become clearer later why this is the case.

Within the context of previously published work on e-nose-based bacterial differentiation, our results partly align but differ markedly in the investigated model system. Sun et al. analyzed planktonic *E. coli*,* S. aureus*, and *P. aeruginosa* using a 34-sensor array reporting accuracies up to 96.15%^55^. Dutta & Dutta achieved up to 100% accuracy for planktonic *E. coli*,* P. aeruginosa*, and *S. aureus*^56^. Dias et al. reported an overall accuracy of 90% for planktonic *E. faecalis*,* S. aureus*,* E. coli*, and *P. aeruginosa*, while Suarez-Cuartin et al. achieved up to 89.2% accuracy in distinguishing *P. aeruginosa* from other pathogens in breath samples^57,58^. These examples illustrate that classification performance strongly depends not only on the applied sensor system and data analysis approach, but also on the experimental conditions, including cultivation and measurement settings. Nevertheless, in comparison with these studies, our findings demonstrate that discrimination remains feasible even under the more complex conditions of biofilm formation in blood.

The number and composition of sensors contributing to predictions varied across folds. For instance, in the decision tree classifier trained on fold 1 for the three-class classification problem, clustering reduced the feature space to five dimensions, combining multiple sensors (i.e., two aggregated clusters and three individual features). Cluster 1 contained 31 features and cluster 2 included 20 features, corresponding to a total of 30 different sensors. The two most informative clusters, defined as those with the highest Shapley values, were used for two-dimensional visualization.

**Fig. 4:**
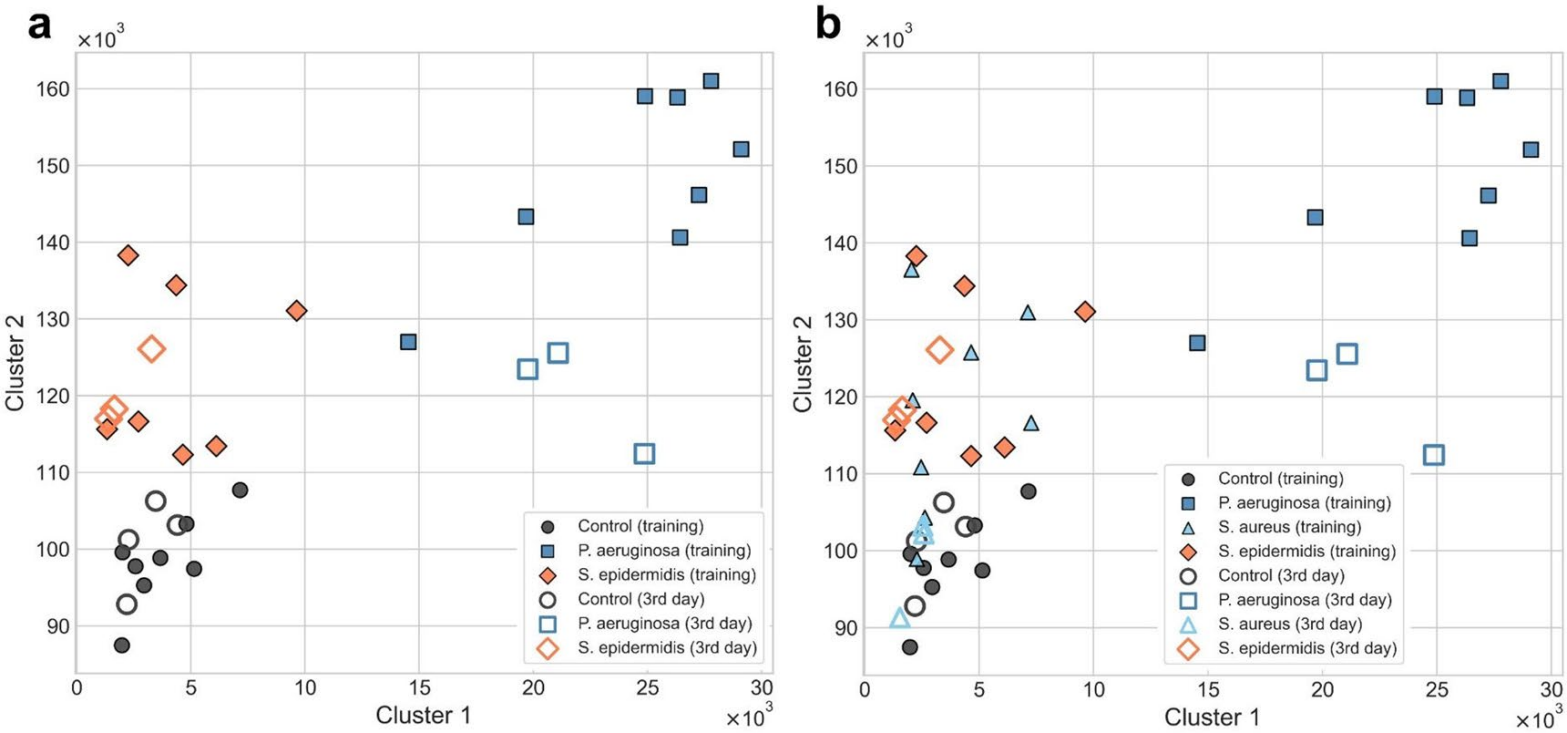
Scatter plots of bacterial samples for the first fold. Solid symbols represent training data, while unfilled symbols indicate test data points. S. aureus exhibits a wide scattering and overlaps with the control or S. epidermidis class, depending on the measurement day. A clear separation of* P. aeruginosa*,* S. epidermidis* and control can be observed (**a**).* S. aureus* scatters more and lies within the control class and* S. epidermidis* class (**b**).

The classes *S. epidermidis*,* P. aeruginosa* and control form well-defined and separable clusters in this space, indicating the presence of an underlying decision function that allows robust class discrimination, as illustrated in Fig. 4a for the first fold (i.e., the third day was used as test day). This demonstrates that a reduction to two dimensions is possible while retaining the most important discriminative information. One can observe that the test samples lie well within the clusters and are consistently classified correctly.

The observed clustering aligns with the findings of Fitzgerald et al., who reported that the VOC profile of *S. epidermidis* was less complex than that of the other species, containing fewer compounds and clustering closer to culture medium. Further, *S. epidermidis* clustered near *S. aureus* in our case, which is consistent with the knowledge of shared key metabolic byproducts. These metabolites are produced by both species, albeit in different quantities and intensities^33^. Overall, these findings indicate that VOC-based separation of these two bacterial species from control samples is feasible when grown in a biofilm-promoting environment.

In the two-dimensional representation, it becomes also evident that the species *S. aureus* lead to markedly different sensor-responses across days. On the first two measurement days, its responses closely resembled those of *S. epidermidis*, whereas on the final day they appeared more similar to the control samples in this features space, as shown in Fig. 4b. This finding is also consistent with literature and likewise shows that *S. aureus* has a markedly different VOC signature than *P. aeruginosa*^32,34^.

Pairwise comparison of feature means among *P. aeruginosa*,* S. epidermidis*, and Control revealed multiple statistically significant differences. Non-parametric permutation tests with 5,000 permutations and α = 0.05 were used. This approach involves random permutations of group labels of the observed data with recomputed statistics to draw statistical inference, especially valuable when only few independent observations are available^59^. The difference between the means was used as test statistic, and p-values were corrected for multiple comparison using the Benjamini-Hochberg procedure. After correction, 79 features differed significantly between *P. aeruginosa* and *S. epidermidis*, 31 features between *S. epidermidis* and Control, and 87 features between *P. aeruginosa* and the Control group. Effect sizes were large (Hedge’s g > 1 for 98%, and > 2 for 78% for the comparisons), and 184 of 197 significant results had corrected p-values below 0.01, indicating robust group separation. All p-values and results are given in Supplementary Table S6.

To investigate the general feasibility of discriminating samples containing bacterial growth from control samples, the task was reformulated as a binary classification problem: all bacterial samples formed one class, retrained against control samples. At this point, it should be noted that *E. faecium* did not provide any sensor responses that differed from the control and could not be distinguished. Therefore, we considered this task without including *E. faecium.* Results (Table 2) show the SVM achieved 95% accuracy, with precision 1.00 for controls and recall 0.97 for biofilms, indicating nearly all bacterial samples were correctly detected, thereby avoiding costly false negatives. Reduced recall for controls reflects their underrepresentation, highlighting that even a few misclassifications strongly affect performance metrics. Increasing control samples in future studies is expected to improve stability and reliability of these estimates. Although the overall accuracy was identical for the kNN and decision tree classifiers, differences were observed in the performance across individual folds. The kNN classifier showed a narrower accuracy range (92.3% to 93.8%), whereas the decision tree exhibited markedly greater variability, with accuracies ranging from 84.6% to 100.0%. This suggests that the kNN model produced more consistent results across different training-test splits. The Receiver Operating Characteristic curves of every classifier and fold are given in Fig. S3 of the Supplementary Materials.

**Table 2:** Classification metrics for each classifier for the binary classification problem ((Biofilm vs. Control) \ E. faecium). Results are reported for the optimized hyperparameter settings. SVM = Support Vector Machine, kNN = k-Nearest-Neighbors, DT = Decision Tree, LR = Logistic Regression, GB = Gradient Boosting, and RF = Random Forest. For each metric, the first value corresponds to the control class and the second to the biofilm class. Metrics were calculated by aggregating all misclassifications across all folds.

	Accuracy	Precision	Recall		F1-Score	
SVM	0.95 ± 0.03	1.00 / 0.94	0.83 / 0.97		0.91 / 0.97	
kNN	0.93 ± 0.01	**1.00** / 0.91	0.75 / **1.00**		0.86 / 0.96	
DT	0.93 ± 0.08	**1.00** / 0.91	0.75 / **1.00**		0.86 / 0.96	
LR	0.91 ± 0.08	0.90 / 0.91	0.75 / 0.97		0.82 / 0.94	
GB	0.93 ± 0.06	0.91 / 0.94	0.83 / 0.97		0.87 / 0.95	
RF	0.93 ± 0.01	1.00 / 0.91	0.75 / 1.00		0.86 / 0.96	

It is further important to note that the overall performance is strongly affected by a single fold. Decrease of performance for some folds reflects pronounced variability between folds and thereby a lower overall cross-validation performance. In strongly imbalanced binary datasets, many commonly reported metrics can be misleading and may overestimate the model performance. As an alternative, the Matthews correlation coefficient (MCC) provides a more informative measure, as it only yields high values when the classification model performs well on both positive and negative samples, taking into account the relative proportions of the classes^60^. We calculated the MCC using all correct and incorrect predictions across folds, offering a fair overview of overall classification performance. For the decision tree and SVM, we obtained an MCC of approximately 0.83 and 0.89, respectively. Overall, the presented binary classification task excluding *E. faecium* represents a simplified best-case scenario to assess the discriminative potential of the sensor-based approach. For clinical applicability, future work must extend this to include all relevant pathogens and evaluate multi-class classification performance under complex and realistic conditions with clinical isolates.

### Consideration of correlations within clusters

Out of the 62 gas sensors, we identified 11 sensors as particularly relevant for the three-class classification task. These sensors were selected in every fold. Notably, this set of chosen sensors includes several neighboring sensors, which in our e-nose system tend to be correlated and exhibit more similar baseline resistance values. Examining for example the two most relevant clusters in fold 1, cluster 2 shows stronger internal correlations than cluster 1. This difference primarily arises from the composition of the clusters: cluster 2 contains only four sensors but multiple features derived from each, whereas cluster 1 comprises 27 sensors, most of which contribute only a single feature. As a result, the features in cluster 2 are inherently more correlated. For each cluster, the feature values were condensed into one value and used as input for the models, reducing the effect of this initial multicollinearity. Using clusters of neighboring sensors could also offer a key advantage: if an individual sensor fails or produces ‘abnormal’ responses, its influence on predictions could be mitigated.

### Quantifying sensor influence in model decision-making

To interpret classifier decisions and identify the most informative sensors, we performed a SHAP analysis. These values quantify the impact of each feature by considering its contribution across all possible combinations of features, providing a measure of feature importance^42^. By linking the important features then back to their corresponding sensors, we visualized representative sensor response curves to illustrate characteristic patterns captured by the models.

Based on the mean absolute Shapley values for the decision tree classifier in the first fold of the three-class problem, the classification decisions were exclusively driven by two clusters. Cluster 2 did not contribute to the predictions of *P. aeruginosa* samples, but showed the same contributions for *S. epidermidis* and the *control* class (0.34). This suggests that the cluster captured more general dynamic behavior common to bacterial classes, rather than species-specific information. The cluster consisted exclusively of derived features from four sensors, including differences, derivatives, absolute values, and their variants. On the other hand, cluster 1 was mainly driven by features describing the initial response slope and the skewness of the signal distribution across the sample exposure and recovery phase. The skewness reflected the balance between response to sample air and subsequent regeneration behavior. This cluster showed the strongest contribution to *P. aeruginosa* predictions (0.44), compared to *S. epidermidis* (0.25) and *control* (0.19). This indicates that cluster 1 encodes species-related signal dynamics. Similar patterns were observed across all folds, but with variations in concrete cluster composition and contribution. Overall, cluster 1 primarily drove discrimination between the two bacterial species, whereas cluster 2 mainly separated control from bacterial samples (see also Fig. 4). Notably, species discrimination required information from a broader set of sensors, whereas control separation relied on a smaller subset.

In contrast to the decision tree classifier, the kNN model did not use clustered features (see Fig. S4 in Supplementary Materials for Shapley value summary plot). The majority of the features included skewness and sample start slopes of single sensors, suggesting that early dynamic signal behavior and recovery characteristics also played a key role for this model. The distribution of the Shapley values across the three classes indicates that while some features contributed to predictions of all classes, others showed more class-specific importance. Notably, only three features deemed important by the kNN classifier were not selected by the decision tree. This strong agreement shows that both classifiers relied on similar sensor information, indicating that these sensors and features captured genuinely discriminative information rather than being selected by chance.

**Fig. 5:**
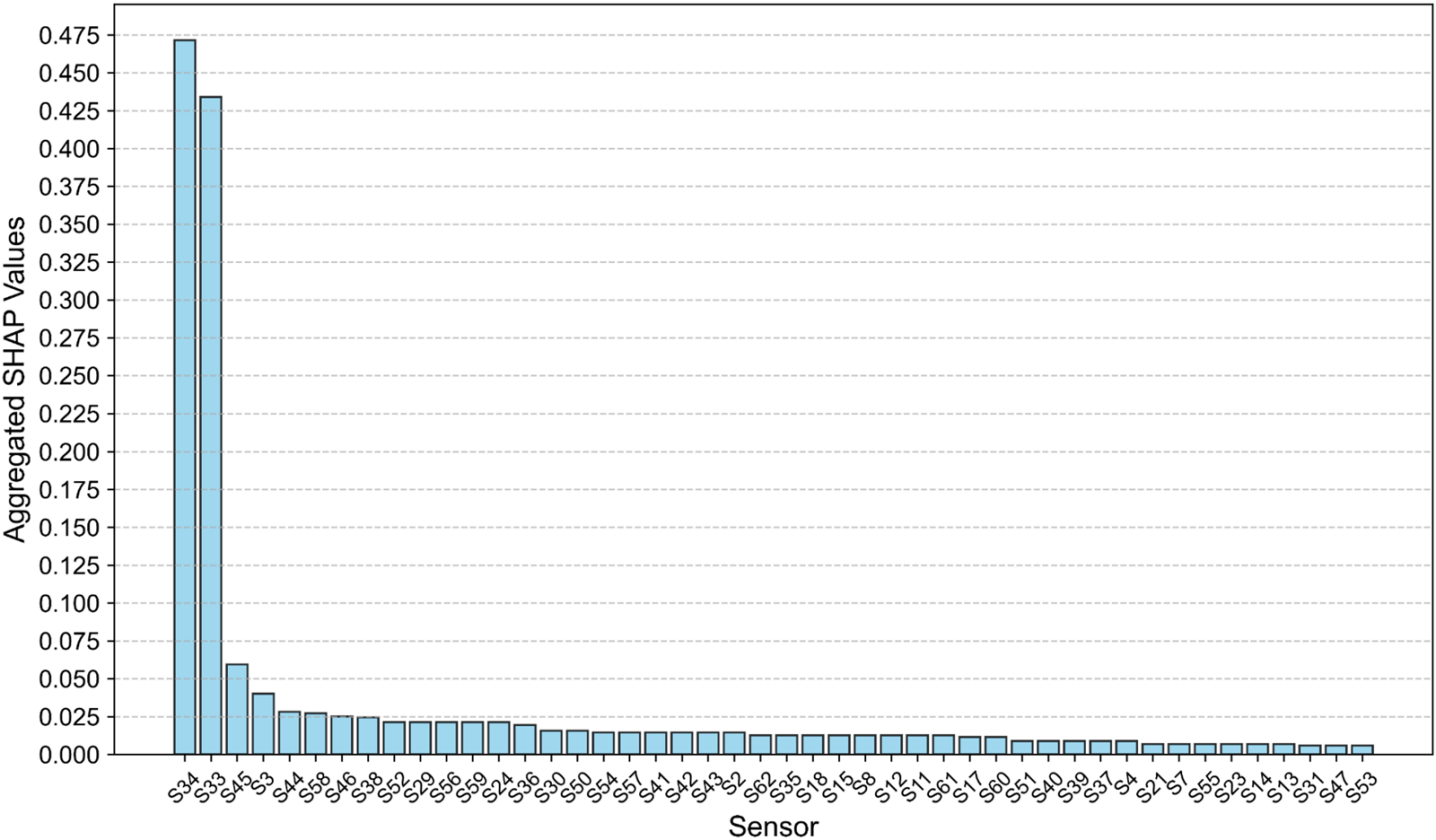
Aggregated Shapley values for each sensor that was used for prediction in at least one fold by the decision tree.

The SHAP values from the decision tree were mapped back from the clusters to individual sensors using weighted attributions. The SHAP value for each cluster was distributed equally among all features within this cluster. After summing up the values for different features of the same sensor within the clusters, we assigned one final value to the sensors. Figure 5 presents these aggregated Shapley values for the decision tree, providing an indication of sensor relevance across cross-validation folds. It should be noted that, due to the summation of the values, these no longer retain their original interpretability. Nevertheless, this metric allows a comparative assessment of which sensors consistently exhibited higher contributions. Two sensors were particularly important for the predictions: sensors 33 and 34. These sensors showed by far the highest values, whereas the remaining sensors exhibited markedly lower contributions. Additionally, several sensors had similar aggregated Shapley values, indicating comparable relevance.

**Fig. 6:**
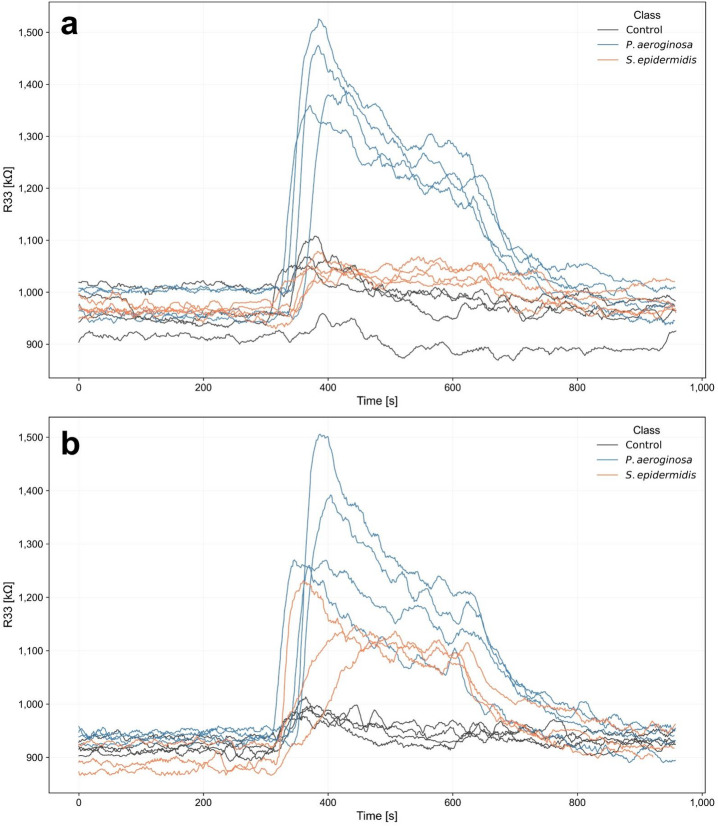
Resistance curves for every sample measurement of control, P. aeruginosa, and S. epidermidis for sensor 33 and the first (**a**) and second (**b**) measurement day. The different biofilm samples induce different sensor responses. The sensor responses were smoothed using a rolling mean with window size of 10.

We further analyzed which features of the response curves (i.e., mean, skewness, recovery slope etc.) of these two sensors contributed most to the model’s decision. For sensor 33 and 34, the decision tree relied on steady-state as well as transient features (i.e., differences, means, derivatives and slopes). Visual inspection of the raw sensor response curves for two different days confirmed these findings (Fig. 6a and b). In particular, the response curves of these two sensors exhibited species-specific shapes, with pronounced differences in the features identified as most relevant by the models. Nevertheless, it is also evident that for *S. epidermidis*, the sensor response shows a varying curve shape. The agreement between model-based relevance and visually identifiable sensor response patterns demonstrates that the workflow provides a transparent and interpretable approach, leading to classification models whose decisions can be meaningfully related to underlying sensor behavior. Further, this supports the conclusion that the discrimination of bacterial cultures that can contain both planktonic and biofilm-associated cells is indeed driven by differently shaped response curves of the sensors. A visualization of these important features is given in Fig. S2 in the Supplementary Materials.

This also shows that relevant information for this classification task is not confined to steady-state features, such as signal differences, but is also captured by transient characteristics, including slopes and derivatives. As reported in literature, such dynamic features can provide complementary and important discriminative information, highlighting their importance for the analysis^27^. It is clear that the plateau resistance values were partly not robust, but the slopes differed markedly, revealing a pronounced distinction between *P. aeruginosa* and the other classes.

**Fig. 7:**
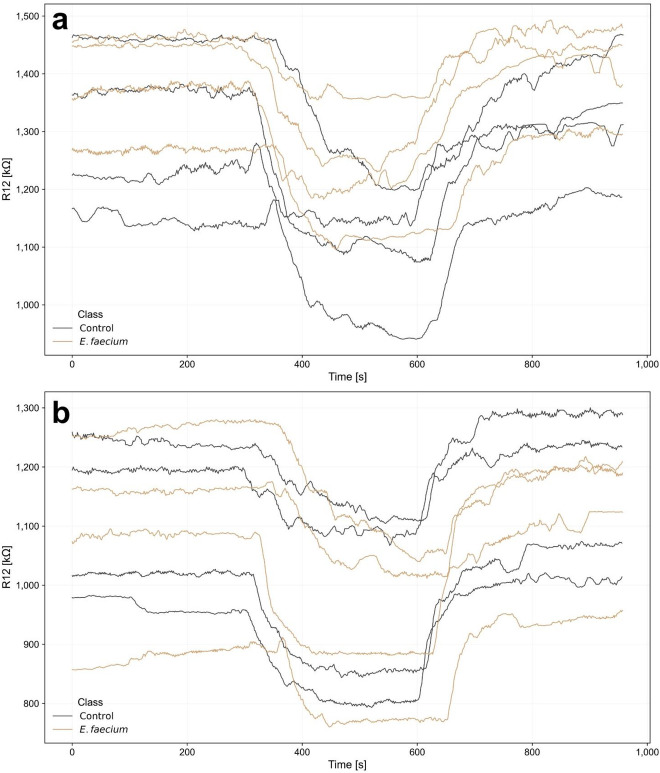
Resistance curves for every sample measurement of control and E. faecium for sensor 12 and the first (**a**) and second (**b**) measurements day. The different biofilm samples induce similar sensor responses. The sensor responses were smoothed using a rolling mean with window size of 10.

Visual inspection of the raw sensor data also indicated substantial similarity between control measurements and *E. faecium* samples, with no consistent characteristic differences. This is exemplified in Fig. 7a and b for the first and second measurement day and sensor 12, which was ranked highest during feature selection. This suggests that even the sensor that was assumed as being most informative captured limited discriminative information for this comparison. It also becomes evident that the sensor responses are highly heterogeneous and vary both between and within days. As a result, the response curves lack consistent shapes and cannot be described by robust features that generalize across days.

### Limitations and outlook

Different limitations must be considered when interpreting these findings. First, the presented study employed a modified Lubbock chronic wound pathogenic biofilm model to investigate biofilm formation under controlled conditions. While the model reflects clinically relevant conditions such as shear stress and polymer-based surface, it remains a simplified in vitro system that cannot fully replicate the complexity of chronic wound environments. Aspects like host immune responses, spatial variability, and dynamic nutrient gradients should be kept in mind. The use of polypropylene tips as an adhesion surface offers a clinically relevant approximation of biofilm formation on polymer-based medical materials. Nevertheless, real clinical materials exhibit a wider range of surface chemistries, geometries, and aging effects, which may influence bacterial attachment and biofilm maturation. Despite these limitations, the wound model offers a controlled and reproducible method to study both planktonic and biofilm-related VOCs. Based on our study, the next step could also include measuring planktonic controls to analyze the difference between volatiles emitted from the biofilm and from planktonic cells under similar growth conditions. Besides these points, the sample size was limited, restricting the robustness of statistical estimates and possibly underestimation of classification performance. On the other hand, we also showed the presence of sample variability, which must be considered to avoid misconclusions about classifier performance. Second, the pronounced day-to-day variability influenced sensor responses and model performance, underscoring the sensitivity of gas sensor measurements to temporal and experimental conditions. In particular, *S. aureus* samples exhibited a broad spread across days, which had an impact on robust classification and suggests substantial variability in volatile emission patterns under the tested conditions. Although no formal drift analysis was performed, measurements were conducted across multiple experimental days, capturing day-to-day variability and thereby reflecting the practical influence of short-term drift. Nevertheless, this does not allow for a quantitative assessment of long-term sensor stability or systematic signal drift, which may affect measurements in prolonged or clinical use. Future work should therefore focus on characterizing isolated sensor drift over extended time period and evaluating calibration and correction strategies to ensure robustness in longitudinal and clinical applications. Such approaches would be essential to improve the reliability and translational potential of the sensing system, particularly in scenarios requiring repeated measurements. As this study focused on the general feasibility and exploration of differentiating biofilm-forming species in blood, sensor drift could not be isolated from biological variability, which is a prerequisite for systematically comparing correction methods. An up-to-date review of drift compensation algorithms for gas sensor arrays is provided by Li et al.^61^. The focus of this review lies on more advanced methods, including deep learning and transfer learning, reflecting the ongoing development of drift correction methodologies in this field. Together, these factors highlight the need for larger datasets with repeated measurements across an extended time period to reliably assess generalizability and biological variability.

A limitation of this study is that biofilm formation was not directly validated using established biofilm characterization methods such as crystal violet staining, microscopy, or viable cell quantification. Consequently, the findings should be interpreted as evidence of bacterial growth under biofilm-promoting conditions rather than detailed characterization of biofilm structure and maturity. Future studies should thus include quantitative biofilm characterization methods to better understand how biofilm structure and biomass relate to VOC signature variability and sensor-based classification performance. Future work may further incorporate GC-MS to validate e-nose signatures and link individual VOCs and metabolites to underlying sensor response patterns. This would support targeted discovery of candidate biomarkers^62–64^. Chen et al. used headspace solid-phase microextraction coupled with GC-MS to differentiate pathogenic species and to assign specific biomarkers, such as 3-methylbutanal and 3-methylbutanoic acid for *S. aureus*^63^. Boots et al. similarly demonstrated that headspace GC-MS reliably identifies microorganisms from their VOC profiles^62^. Further, metabolites originating from biofilms of *P. aeruginosa*, like 1-undecene and 2-undecanone, could be detected in concentrations in the low nanomolar range using a GC-MS based method^64^. As GC-MS studies in literature demonstrated that growth medium and incubation temperature determine the emitted VOC spectrum, these measurements would allow to interpret the day-to-day and species-dependent variability observed in our blood-based environment^37^. This could also resolve structurally overlapping profiles, particularly for *E. faecium* and highly variable *S. aureus*, and guiding biomarker selection as well as sensor array improvements.

As demonstrated by Bean et al. for *P. aeruginosa*, clinical isolates can exhibit substantial variability in their volatile profiles, with certain compounds consistently detected across all isolates, while others appear to be isolate-specific^65^. Their findings suggest the existence of a core volatilome shared among strains, alongside additional volatiles that have not previously been associated with this species. This highlights the importance of considering strain-level heterogeneity with real clinical isolates for future studies to better characterize variability in VOC patterns.

Despite the limitations of this study, the results provide initial evidence that discrimination of certain bacterial species is feasible even under clinically relevant conditions, including biofilm formation and the presence of blood. These findings also offer an indication of the potential of e-noses for biofilm identification.

## Conclusion

This study was designed as a proof-of-principle to assess whether an e-nose can differentiate between bacterial species even in the presence of biofilm formation and blood components in the medium. These conditions reflect clinically relevant complexities, making successful discrimination a meaningful indicator of the potential. We demonstrated that classification of control samples, *P. aeruginosa* and *S. epidermidis* was achievable with an accuracy of more than 94%. Nevertheless, *E. faecium* produced sensor responses highly similar to those of the control and could not be reliably distinguished. These biological limitations likely exceed the sensitivity of the employed sensor array. In addition, *S. aureus* showed pronounced day to day variability in its sensor response patterns, preventing stable and consistent classification across measurement days. Beyond classification performance, we illustrated a workflow for dimensionality reduction in high dimensional, small sample size datasets with highly correlated gas sensor data, producing interpretable features for which contributions can be quantified using Shapley values. We showed that only a subset of sensors and features is required to achieve discrimination. At the same time, the study reveals constraints, particularly the pronounced influence of temporal variability. Differences between measurement days affected model performance, emphasizing the need to account for day-to-day variability and sampling bias when training and evaluating classifiers. As it is known from literature, volatile compounds are closely linked to biofilm physiology and dynamics, making e-noses promising tools for their detection and monitoring. This motivates future studies to investigate sensor responses over time to capture biofilm dynamics, enhancing assessment of clinical relevance. As this dataset is limited in size and temporal coverage, future work should also focus on expanding the dataset with more measurements and bacterial species, which may enhance both predictive performance and biological insights.

## Supplementary Information

Below is the link to the electronic supplementary material.

Supplementary Material 1

## Data Availability

The data generated and analyzed during this study are included in the Supplementary Material files.

## Code availability

The python code used for loading the measurements, preprocessing the data and model training are provided as Supplementary File 2.
