# Supplementary material for: Data-driven exploration of electronic nose technology to differentiate bacteria in blood cultures under biofilm-promoting conditions
Source: Sci Rep. 2026 Jul 10;16:21641. doi: 10.1038/s41598-026-62071-8 (PMC13354572; doi:10.1038/s41598-026-62071-8)
Supplement: Supplementary file 1 — Supplementary Material 1 [file 41598_2026_62071_MOESM1_ESM.zip › Supplementary Figures.pdf]

Supplementary Figures

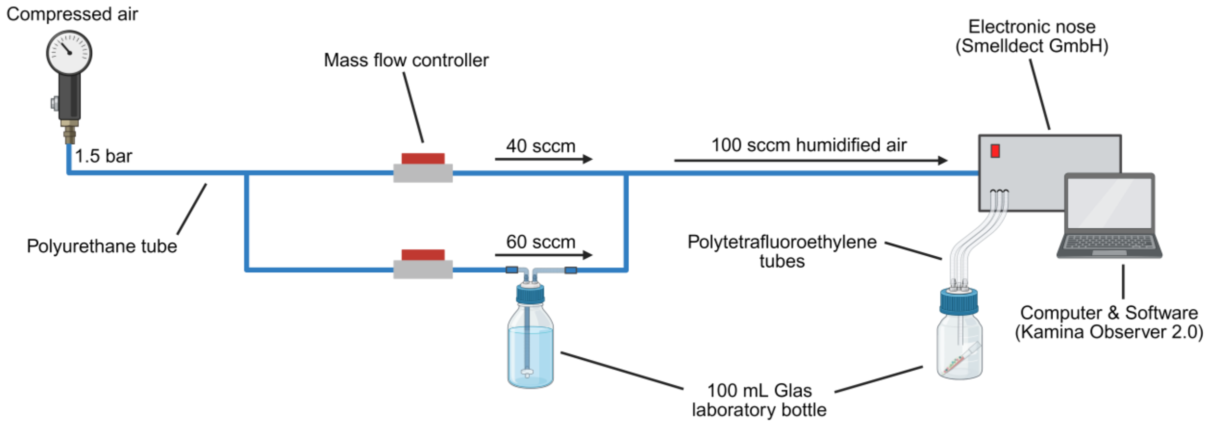

Figure S1: Schematic drawing of the experimental setup. Compressed air served as carrier gas, with mass flow controllers regulating both flow rates and humidity. The closed system ensures that all measurements were conducted independently of ambient air conditions. Created in BioRender, Pein-Hackelbusch, M. (2026) https://BioRender.com/wxim7cz.

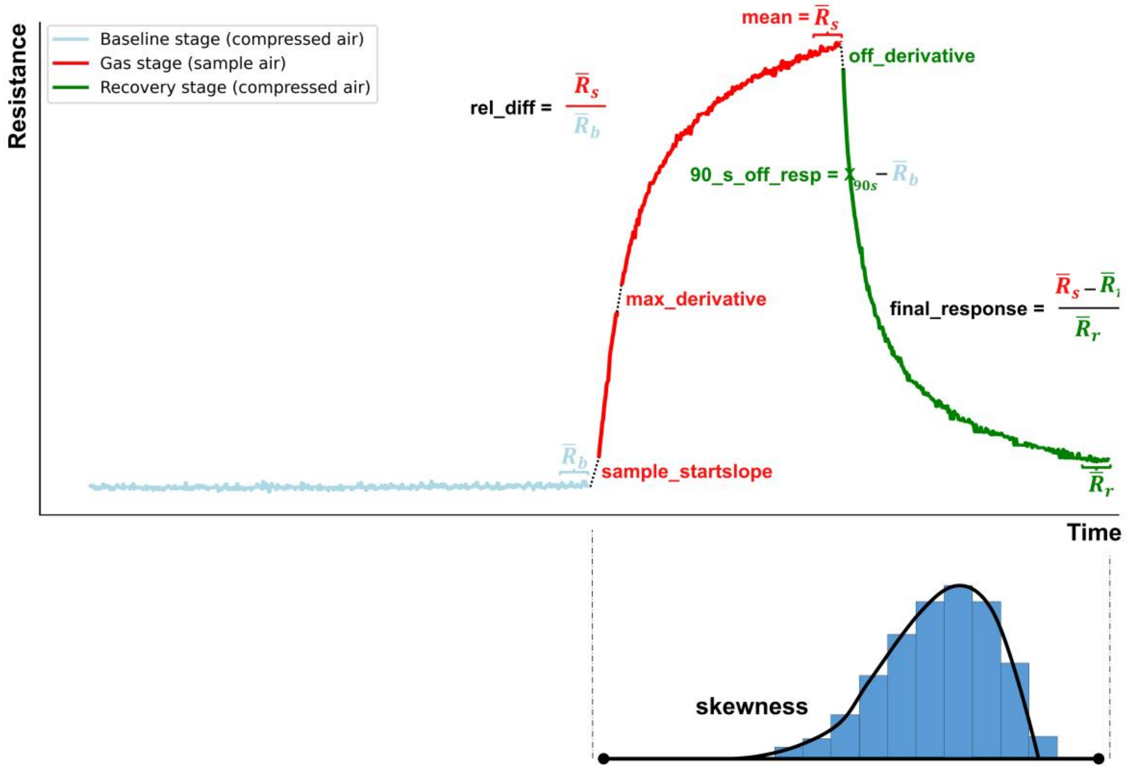

Figure S2: Visualization of a resistance curve of one sensor measured for a single sample across the three stages baseline, sample air exposure, and recovery, each highlighted in a different color. Characteristic features extracted from the signal are annotated, illustrating key parameters that contributed to the classification process.

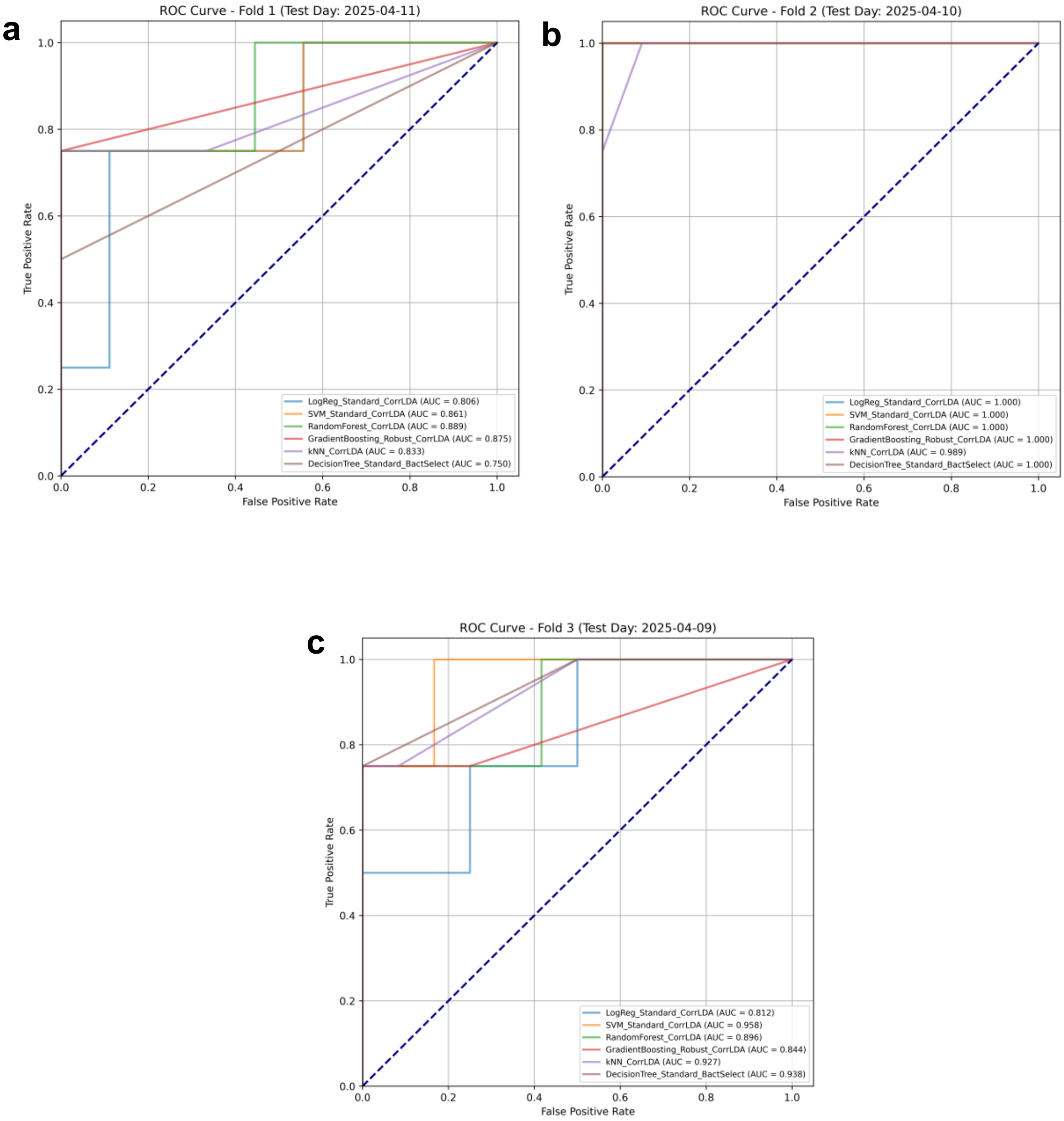

Figure S3: Receiver operating characteristic (ROC) curves of the different classifiers evaluated across the three cross-validation folds. Panels a), b) and c) corrspond to fold 1, fold 2, and fold 3, respectively. Each curve illustrates the trade-off between the True Positive Rate and the False Positive Rate for the respective classifier within the corresponding fold.

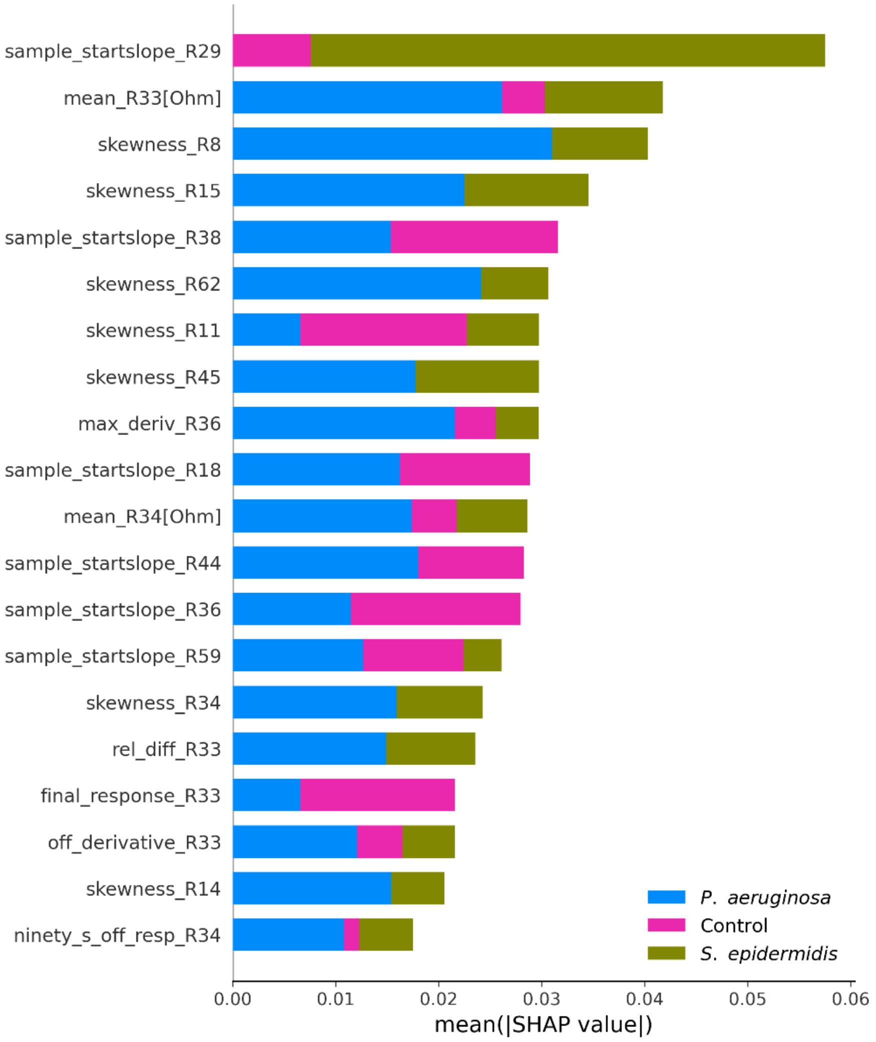

Figure S4: Mean absolute SHAP values for the kNN classifier in fold 1, including 20 individual features. The values represent the average impact of each feature on the model output, thereby indicating their relative importance. Due to the non-deterministic nature of these estimations for kNN, the values should be interpreted as indicative rather than exact measures of feature relevance. Features are ranked according to their mean absolute values, indicating their overall contribution to model predictions across all classes.
