# Supplementary material for: Data-driven exploration of electronic nose technology to differentiate bacteria in blood cultures under biofilm-promoting conditions
Source: Sci Rep. 2026 Jul 10;16:21641. doi: 10.1038/s41598-026-62071-8 (PMC13354572; doi:10.1038/s41598-026-62071-8)
Supplement: Supplementary file 1 — Supplementary Material 1 [file 41598_2026_62071_MOESM1_ESM.zip › Supplementary File 2.html]

Code für das Biofilmpaper


In [ ]:

```
"""
The pipeline automatically runs a variety of feature selection methods with different classifiers via leave-one-day out CV.

Author: Jonas Eimler (OWL University of Applied Sciences and Arts, 32657 Lemgo, Germany)
Contributors: Julius Wörner (Institute for Life Science Technologies (ILT.NRW), OWL University of Applied Sciences and Arts, 32657 Lemgo, Germany)
Created: November 2025
"""


import warnings
import numpy as np
import pandas as pd
from sklearn.svm import SVC
from sklearn.base import clone
from sklearn.pipeline import Pipeline
from itertools import combinations, product
from scipy.spatial.distance import squareform
from sklearn.preprocessing import LabelEncoder
from sklearn.tree import DecisionTreeClassifier
from sklearn.model_selection import GridSearchCV
from sklearn.neighbors import KNeighborsClassifier
from sklearn.linear_model import LogisticRegression
from scipy.cluster.hierarchy import linkage, fcluster
from sklearn.base import BaseEstimator, TransformerMixin
from sklearn.feature_selection import SelectKBest, f_classif
from sklearn.preprocessing import RobustScaler, StandardScaler
from sklearn.discriminant_analysis import LinearDiscriminantAnalysis
from statsmodels.stats.outliers_influence import variance_inflation_factor
from sklearn.ensemble import RandomForestClassifier, GradientBoostingClassifier
from sklearn.metrics import accuracy_score, classification_report, confusion_matrix


# warnings.filterwarnings('ignore')


# ====================================================================
# Load the data and preprocess it
# ====================================================================

# Read the CSV with extracted features
features_df = pd.read_csv(r'D:\XX\extracted_features.csv', sep=' ')

# Renaming classes globally (mapping all class names to bacteria categories)
def map_class_names(class_name):
    """
    Maps the more detailed class names to their bacterial species.
    """
    prefixes = ['Efa', 'Kontrolle', 'Pae', 'Sau', 'Sepi']
    for prefix in prefixes:
        if class_name.startswith(prefix):
            if class_name.startswith('Kontrolle'):
                return 'Control'
            else:
                return prefix
    return class_name 

features_df['Class'] = features_df['Class'].apply(map_class_names)

# ====================================================================
# This is the class for selecting the features
# ====================================================================

class BasePairwiseBacteriaSelector(BaseEstimator, TransformerMixin):
    """    
    This class
    1. Performs pairwise class comparisons using SelectKBest from sklearn
    2. Collects the union of selected features across all pairwise comparisons
    3. Finally applys various strategies for final feature selection
    
    Attributes:
        k (int): Number of top features to select in each pairwise comparison
        score_func (callable): Scoring function for SelectKBest (default: f_classif)
        corr_threshold (float): Correlation threshold for clustering (if applicable)
        feature_names_in_ (list): Names of input features
        classes_ (ndarray): Dynamically extracted unique classes from the target variable
    """
    
    def __init__(self, k=20, score_func=f_classif, corr_threshold=0.8):
        """
        Initialize the base selector.
        """
        self.k = k
        self.score_func = score_func
        self.corr_threshold = corr_threshold
        self.feature_names_in_ = None
        self.classes_ = None 
    
    def _prepare_data(self, X, y):
        """
        Convert input data to numpy arrays and extract feature names.
        Args:
            X (array-like or DataFrame): Input features of shape (n_samples, n_features)
            y (array-like or Series): Target labels of shape (n_samples,)
            
        Returns:
            tuple: (X_array, y_array, feature_names) where:
                - X_array is a 2D numpy array of features
                - y_array is a 1D numpy array of labels
                - feature_names is a list of feature names
        """
        # Extract feature names from DataFrame columns or genereate names when applicable
        if hasattr(X, 'columns'):
            feature_names = X.columns.tolist()
            X_array = X.values
        else:
            feature_names = [f'feature_{i}' for i in range(X.shape[1])]
            X_array = np.asarray(X)
        
        # Convert y to 1D numpy array
        if isinstance(y, (pd.DataFrame, pd.Series)):
            y_array = y.values.ravel() if isinstance(y, pd.DataFrame) else y.values
        else:
            y_array = np.asarray(y).ravel()
        
        return X_array, y_array, feature_names
    
    def _pairwise_select_k_best(self, X_array, y_array):
        """
        Perform SelectKBest on all pairwise combinations of classes

        The function
        1. Dynamically identifies all unique classes in y_array
        2. Iterates through all pairwise combinations of these classes
        3. For each pair, extracts samples belonging to those two classes
        4. Runs SelectKBest to find the k most discriminative features
        5. Collects all selected features and their scores
        
        Args:
            X_array (ndarray): Feature matrix of shape (n_samples, n_features)
            y_array (ndarray): Label array of shape (n_samples,)
            
        The function returns:
            tuple: (union_selected_features, feature_scores) where:
                - union_selected_features is a set of all selected feature names
                - feature_scores is a dict mapping feature names to lists of scores
        """
        union_selected_features = set()
        feature_scores = {}
        
        self.classes_ = np.unique(y_array)

        
        # Iterate through all pairwise combinations of the actual classes in y_array
        for class1, class2 in combinations(self.classes_, 2):
            # Create binary mask for current pair of classes
            mask = (y_array == class1) | (y_array == class2)
            
            # Skip if insufficient samples
            if mask.sum() < 2:
                continue
            
            # Extract samples for this pairwise comparison
            X_combo = X_array[mask]
            y_combo = y_array[mask]
            
            try:
                # Run SelectKBest on this comparison
                selector = SelectKBest(
                    score_func=self.score_func, 
                    k=min(self.k, X_combo.shape[1])
                )
                selector.fit(X_combo, y_combo)
                
                # Get the boolean mask of selected features
                feature_mask = selector.get_support()
                combo_indices = np.where(feature_mask)[0]
                combo_features = [self.feature_names_in_[i] for i in combo_indices]
                
                # Add selected features to the union
                union_selected_features.update(combo_features)
                
                # Store scores for each feature
                for feature, score in zip(combo_features, selector.scores_[feature_mask]):
                    feature_scores.setdefault(feature, []).append(score)
                    
            except Exception as e:
                print(f"    Warning: SelectKBest failed for {class1} vs {class2}: {e}")
                continue
        
        return union_selected_features, feature_scores
    

# ====================================================================
# Now the classes for transforming the features for the models
# ====================================================================

class BacteriaFeatureSelector(BasePairwiseBacteriaSelector):
    """
    Pairwise feature selector that collects the union of all features selected across pairwise class comparisons.
    1. For each pair of bacteria classes, run SelectKBest to find top k features
    2. Collect the union of all selected features
    3. Transform data by selecting only these features (but without further reduction)
    """
    
    def __init__(self, k=20, score_func=f_classif):
        """
        Initialize the selector.
        
        Args:
            k (int): Number of top features to select in each pairwise comparison
        """
        super().__init__(k=k, score_func=score_func, corr_threshold=None)
        self.selected_features_ = None
        self.feature_scores_ = {}
        self.selected_indices_ = None
        
    def fit(self, X, y):
        """
        Fit the selector by performing pairwise SelectKBest and collecting the union.
        
        Args:
            X: Training features
            y: Target labels
            
        Returns:
            self: Fitted transformer
        """
        
        # Convert input data to numpy arrays and extract feature names
        X_array, y_array, self.feature_names_in_ = self._prepare_data(X, y)
        
        # Perform pairwise SelectKBest to collect features
        union_selected_features, feature_scores = self._pairwise_select_k_best(X_array, y_array)
        
        # Calculate average score for each feature across all pairwise comparisons
        self.feature_scores_ = {f: np.mean(s) for f, s in feature_scores.items()}
        self.selected_features_ = sorted(list(union_selected_features))
        
        # Save indices of selected features
        self.selected_indices_ = [
            i for i, name in enumerate(self.feature_names_in_) 
            if name in self.selected_features_
        ]
        
        return self
    
    def transform(self, X):
        """
        Transform X by selecting only the fitted features.
        
        Args:
            X: Data to transform
            
        Returns:
            ndarray: Transformed data with only selected features
        """
        if self.selected_indices_ is None:
            raise ValueError("Fit must be run before transform!")
        
        # Convert to numpy array if necessary
        X_array = X.values if hasattr(X, 'values') else np.asarray(X)
        
        # Extract only the selected feature columns
        return X_array[:, self.selected_indices_]


class SelectKBestPairwiseCorrAggregator(BasePairwiseBacteriaSelector):
    """
    Feature selector with correlation-based clustering and aggregation.
    This selector reduces the selected feature space by looking at correlations.
    
    1. Performing pairwise SelectKBest to collect initial features (same as before)
    2. Clustering highly correlated features using hierarchical clustering
    3. Aggregating features within each cluster by averaging them --> one cluster becomes one feature
    
    Attributes:
        cluster_composition_ (dict): Maps output feature names to input features
    """
    
    def __init__(self, k=20, score_func=f_classif, corr_threshold=0.8):
        """
        Initialize the selector.
        
        Args:
            k (int): Number of top features to select in each pairwise comparison
            score_func (callable): Scoring function for SelectKBest
            corr_threshold (float): Correlation threshold for clustering
        """
        super().__init__(k=k, score_func=score_func, corr_threshold=corr_threshold)
        self.initial_indices_ = None
        self.feature_clusters_ = None
        self.aggregation_matrix_ = None
        self.feature_names_out_ = None
        self.cluster_composition_ = {}

    def fit(self, X, y):
        """
        Fit the selector through pairwise selection, clustering, and aggregation.
        
        This method performs all three main steps mentioned above.
        
        Args:
            X: Training features
            y: Target labels
        """
        # Prepare data
        X_array, y_array, self.feature_names_in_ = self._prepare_data(X, y)
        
        # Step 1: Pairwise SelectKBest to create initial feature set
        union_selected_features, _ = self._pairwise_select_k_best(X_array, y_array)
        
        # Get indices and data for selected features
        selected_feature_names = sorted(list(union_selected_features))
        self.initial_indices_ = [self.feature_names_in_.index(f) for f in selected_feature_names]
        X_selected = X_array[:, self.initial_indices_]
        
        # Step 2: Correlation-based clustering
        X_selected_df = pd.DataFrame(X_selected, columns=selected_feature_names)
        correlation_matrix = X_selected_df.corr().fillna(0)
        
        # Convert correlation to distance metric: high correlation means low distance
        distance_matrix = 1 - np.abs(correlation_matrix.values)
        np.fill_diagonal(distance_matrix, 0)
        
        # Hierarchical clustering using 'complete' (which is maximum linkage)
        linkage_matrix = linkage(squareform(distance_matrix), method='complete')
        # Cut dendrogram at threshold to form clusters
        clusters = fcluster(linkage_matrix, t=1 - self.corr_threshold, criterion='distance')
        
        # Group features by cluster ID
        feature_clusters = {}
        for feature, cluster_id in zip(selected_feature_names, clusters):
            feature_clusters.setdefault(cluster_id, []).append(feature)
        self.feature_clusters_ = feature_clusters
        
        # Step 3: Create aggregation matrix
        n_selected = len(selected_feature_names)
        n_output = len(feature_clusters)
        self.aggregation_matrix_ = np.zeros((n_selected, n_output))
        
        self.feature_names_out_ = []
        
        for i, (cluster_id, features) in enumerate(sorted(feature_clusters.items())):
            # Get indices of features in this cluster
            cluster_indices = [selected_feature_names.index(f) for f in features]
            
            # Set equal weights for averaging (1/n for each feature in cluster)
            self.aggregation_matrix_[cluster_indices, i] = 1.0 / len(features)
            
            # Name the output feature
            if len(features) > 1:
                output_name = f"aggregated_cluster_{cluster_id}"
                self.cluster_composition_[output_name] = features
            else:
                # Single-feature clusters keep their original name
                output_name = features[0]
                self.cluster_composition_[output_name] = features
                
            self.feature_names_out_.append(output_name)
        
        return self

    def transform(self, X):
        """
        Transform X by aggregating correlated features.
        
        Args:
            X: Data to transform
            
        Returns:
            ndarray: Transformed data with aggregated features
        """
        if self.aggregation_matrix_ is None:
            raise ValueError("Fit must be called before transform")
        
        X_array = X.values if hasattr(X, 'values') else np.asarray(X)
        
        # Extract the initially selected features
        X_selected = X_array[:, self.initial_indices_]
        # Apply aggregation matrix
        X_aggregated = X_selected @ self.aggregation_matrix_
        
        return X_aggregated
    


class SelectKBestPairwiseCorrDropper(BasePairwiseBacteriaSelector):
    """
    Feature selector with correlation-based clustering and 'first-feature selection'.

    1. Pairwise SelectKBest to collect initial features
    2. Correlation-based clustering
    3. Keep only the first feature from each cluster
    """
    
    def __init__(self, k=20, score_func=f_classif, corr_threshold=0.8):
        """
        Initialize the selector.
        
        Args:
            k (int): Number of top features to select in each pairwise comparison
            score_func (callable): Scoring function for SelectKBest
            corr_threshold (float): Correlation threshold for clustering
        """
        super().__init__(k=k, score_func=score_func, corr_threshold=corr_threshold)
        self.feature_clusters_ = None
        self.final_indices_ = None
        self.feature_names_out_ = None

    def fit(self, X, y):
        """
        Fit the selector by clustering and keeping first feature per cluster.
        
        Args:
            X: Training features
            y: Target labels
            
        Returns:
            self: Fitted transformer
        """
        # Prepare data
        X_array, y_array, self.feature_names_in_ = self._prepare_data(X, y)
        
        # Step 1: Pairwise SelectKBest to create initial feature pool
        union_selected_features, _ = self._pairwise_select_k_best(X_array, y_array)
        
        # Get selected feature data
        selected_feature_names = sorted(list(union_selected_features))
        initial_indices = [self.feature_names_in_.index(f) for f in selected_feature_names]
        X_selected = X_array[:, initial_indices]
        
        # Step 2: Correlation-based clustering 
        X_selected_df = pd.DataFrame(X_selected, columns=selected_feature_names)
        correlation_matrix = X_selected_df.corr().fillna(0)
        
        distance_matrix = 1 - np.abs(correlation_matrix.values)
        np.fill_diagonal(distance_matrix, 0)
        
        linkage_matrix = linkage(squareform(distance_matrix), method='complete')
        clusters = fcluster(linkage_matrix, t=1 - self.corr_threshold, criterion='distance')
        
        feature_clusters = {}
        for feature, cluster_id in zip(selected_feature_names, clusters):
            feature_clusters.setdefault(cluster_id, []).append(feature)
        self.feature_clusters_ = feature_clusters
        
        # Step 3: Select the first feature per cluster
        kept_features = []
        for cluster_id, features in sorted(feature_clusters.items()):
            # Always keep the first feature (sorted alphabetically)
            kept_features.append(features[0])
        
        self.feature_names_out_ = kept_features
        self.final_indices_ = [self.feature_names_in_.index(f) for f in kept_features]
        
        
        return self

    def transform(self, X):
        """
        Transform X by selecting only the kept features.
        
        Args:
            X: Data to transform
            
        Returns:
            ndarray: Transformed data with only kept features
        """
        if self.final_indices_ is None:
            raise ValueError("fit must be called before transform!")
        
        X_array = X.values if hasattr(X, 'values') else np.asarray(X)
        return X_array[:, self.final_indices_]


class SelectKBestCorrelationLDA(BasePairwiseBacteriaSelector):
    """
    Feature selector with correlation clustering and Linear Discriminant Analysis.
    
    Applies dimensionality reduction using LDA.
    
    1. Pairwise SelectKBest to collect initial features
    2. Correlation-based clustering
    3. For each multi-feature cluster, apply LDA to reduce dimensions and use the latent variables as new features
    4. Single-feature clusters are kept the same
    """
    
    def __init__(self, k=20, score_func=f_classif, corr_threshold=0.8, max_lda_components=3):
        """
        Initialize the selector.
        
        Args:
            k (int): Number of top features to select in each pairwise comparison
            score_func (callable): Scoring function for SelectKBest
            corr_threshold (float): Correlation threshold for clustering
            max_lda_components (int): Maximum LDA components to extract per cluster
        """
        super().__init__(k=k, score_func=score_func, corr_threshold=corr_threshold)
        self.max_lda_components = max_lda_components
        self.selected_features_ = None
        self.avg_feature_scores_ = None
        self.feature_clusters_ = None
        self.lda_results_ = None
        self.final_feature_names_ = None
    
    def fit(self, X, y):
        """
        Fit the selector using pairwise selection, clustering, and LDA.
        
        Args:
            X: Training features
            y: Target labels
            
        Returns:
            self: Fitted transformer
        """
        # Prepare data
        X_array, y_array, self.feature_names_in_ = self._prepare_data(X, y)
        
        # Pairwise SelectKBest and score calculation
        union_selected_features, feature_scores = self._pairwise_select_k_best(X_array, y_array)
        
        # Calculate average scores (will be used for weighted fallback if LDA fails)
        self.avg_feature_scores_ = {f: np.mean(s) for f, s in feature_scores.items()}
        selected = list(self.avg_feature_scores_.keys())
        self.selected_features_ = selected
        
        selected_indices = [self.feature_names_in_.index(f) for f in selected]
        X_selected = X_array[:, selected_indices]
        
        # Correlation-based clustering
        features_df = pd.DataFrame(X_selected, columns=selected)
        corr_matrix = features_df.corr().fillna(0)
        
        distance_matrix = 1 - np.abs(corr_matrix.values)
        np.fill_diagonal(distance_matrix, 0)
        linkage_matrix = linkage(squareform(distance_matrix), method='complete')
        clusters = fcluster(linkage_matrix, t=1 - self.corr_threshold, criterion='distance')
        
        cluster_map = {}
        f_cols = list(corr_matrix.columns)
        for feat, cl_id in zip(f_cols, clusters):
            cluster_map.setdefault(cl_id, []).append(feat)
        self.feature_clusters_ = cluster_map
        
        # Apply LDA to each cluster
        lda_results = {}
        new_features_list = []
        new_feature_names = []
        
        n_classes = len(np.unique(y_array))
        
        for cl_id, feats in self.feature_clusters_.items():
            if len(feats) < 2:
                # Single-feature cluster: keep as-is
                feat_idx = [selected.index(f) for f in feats]
                new_features_list.append(X_selected[:, feat_idx])
                new_feature_names.extend(feats)
                continue
            
            # Multi-feature cluster: apply LDA
            feat_indices = [selected.index(f) for f in feats]
            X_cluster = X_selected[:, feat_indices]
            
            # Calculate maximum possible LDA components
            min_per_class = min([np.sum(y_array == cls) for cls in np.unique(y_array)])
            max_comps = min(n_classes - 1, len(feats), len(X_cluster), 
                          min_per_class//2, self.max_lda_components)
            final_comps = max(1, max_comps)
            
            try:
                # Fit LDA to this cluster
                lda = LinearDiscriminantAnalysis(n_components=final_comps)
                X_lda = lda.fit_transform(X_cluster, y_array)
                
                new_features_list.append(X_lda)
                for ci in range(final_comps):
                    new_feature_names.append(f'lda_group_{cl_id}_comp_{ci+1}')
                
                # Store LDA model for transform phase
                lda_results[cl_id] = {
                    'lda': lda, 'features': feats, 'n_components': final_comps,
                    'explained_variance_ratio': getattr(lda, 'explained_variance_ratio_', None)
                }
            except Exception as e:
                # Fallback: weighted average if LDA fails
                weights = np.array([self.avg_feature_scores_.get(feat, 1.0) for feat in feats])
                norm_weights = weights / np.sum(weights) if np.sum(weights) > 0 else np.ones_like(weights)/len(weights)
                fallback_feat = (X_cluster * norm_weights).sum(axis=1, keepdims=True)
                new_features_list.append(fallback_feat)
                new_feature_names.append(f'weighted_mean_group_{cl_id}')
        
        # Combine all features into a single array
        self._X_fit_ = np.hstack(new_features_list)
        self.final_feature_names_ = new_feature_names
        self.lda_results_ = lda_results
        self.n_features_out_ = len(new_feature_names)
        self.n_features_in_ = X_array.shape[1]
        
        return self
    
    def transform(self, X):
        """
        Transform X by applying LDA transformations.
        
        Args:
            X: Data to transform
            
        Returns:
            ndarray: Transformed data with LDA components
        """
        X_array = X.values if hasattr(X, 'values') else np.asarray(X)
        
        # Extract selected features
        selected_indices = [self.feature_names_in_.index(f) for f in self.selected_features_]
        X_selected = X_array[:, selected_indices]
        
        # Apply saved transformation for each cluster
        new_features_list = []
        
        for cl_id, feats in self.feature_clusters_.items():
            if len(feats) < 2:
                # Single-feature cluster: keep as-is
                feat_idx = [self.selected_features_.index(f) for f in feats]
                new_features_list.append(X_selected[:, feat_idx])
                continue
            
            feat_indices = [self.selected_features_.index(f) for f in feats]
            X_cluster = X_selected[:, feat_indices]
            
            lda_info = self.lda_results_.get(cl_id, None)
            if lda_info is not None:
                lda = lda_info['lda']
                try:
                    # Apply LDA transformation
                    X_lda = lda.transform(X_cluster)
                    new_features_list.append(X_lda)
                except Exception as e:
                    # Fallback: weighted average
                    weights = np.array([self.avg_feature_scores_.get(feat, 1.0) for feat in feats])
                    norm_weights = weights / np.sum(weights) if np.sum(weights) > 0 else np.ones_like(weights)/len(weights)
                    fallback_feat = (X_cluster * norm_weights).sum(axis=1, keepdims=True)
                    new_features_list.append(fallback_feat)
            else:
                # Fallback if no LDA model was saved
                weights = np.array([self.avg_feature_scores_.get(feat, 1.0) for feat in feats])
                norm_weights = weights / np.sum(weights) if np.sum(weights) > 0 else np.ones_like(weights)/len(weights)
                fallback_feat = (X_cluster * norm_weights).sum(axis=1, keepdims=True)
                new_features_list.append(fallback_feat)
        
        return np.hstack(new_features_list)
    


class SelectKBestPairwiseCorrBestScore(BasePairwiseBacteriaSelector):
    """
    Feature selector with correlation clustering and best-score selection.
    
    1. Pairwise SelectKBest to collect initial features with scores
    2. Calculate average score for each feature across all pairwise comparisons
    3. Correlation-based clustering
    4. Keep only the feature with highest average score from each cluster
    """
    
    def __init__(self, k=20, score_func=f_classif, corr_threshold=0.8):
        """
        Initialize the selector.
        
        Args:
            k (int): Number of top features to select in each pairwise comparison
            score_func (callable): Scoring function for SelectKBest
            corr_threshold (float): Correlation threshold for clustering
        """
        super().__init__(k=k, score_func=score_func, corr_threshold=corr_threshold)
        self.avg_feature_scores_ = None
        self.feature_clusters_ = None
        self.final_indices_ = None
        self.feature_names_out_ = None

    def fit(self, X, y):
        """
        Fit the selector
        
        """
        # Prepare data
        X_array, y_array, self.feature_names_in_ = self._prepare_data(X, y)
        
        # Pairwise SelectKBest with score collection
        union_selected_features, feature_scores = self._pairwise_select_k_best(X_array, y_array)
        
        # Calculate average score for each feature
        self.avg_feature_scores_ = {f: np.mean(s) for f, s in feature_scores.items()}
        
        # Get selected feature data
        selected_feature_names = sorted(list(union_selected_features))
        initial_indices = [self.feature_names_in_.index(f) for f in selected_feature_names]
        X_selected = X_array[:, initial_indices]
        
        # Correlation-based clustering
        X_selected_df = pd.DataFrame(X_selected, columns=selected_feature_names)
        correlation_matrix = X_selected_df.corr().fillna(0)
        
        distance_matrix = 1 - np.abs(correlation_matrix.values)
        np.fill_diagonal(distance_matrix, 0)
        
        linkage_matrix = linkage(squareform(distance_matrix), method='complete')
        clusters = fcluster(linkage_matrix, t=1 - self.corr_threshold, criterion='distance')
        
        feature_clusters = {}
        for feature, cluster_id in zip(selected_feature_names, clusters):
            feature_clusters.setdefault(cluster_id, []).append(feature)
        self.feature_clusters_ = feature_clusters
        
        # Select feature with highest score from each cluster
        kept_features = []
        for cluster_id, features in sorted(feature_clusters.items()):
            if len(features) == 1:
                kept_features.append(features[0])
            else:
                best_feature = max(features, key=lambda f: self.avg_feature_scores_.get(f, -1))
                kept_features.append(best_feature)
        
        self.feature_names_out_ = kept_features
        self.final_indices_ = [self.feature_names_in_.index(f) for f in kept_features]
        
        
        return self

    def transform(self, X):
        """
        Transform X by selecting only the best-scoring features.
        
        Args:
            X: Data to transform
            
        Returns:
            ndarray: Transformed data with selected features
        """
        if self.final_indices_ is None:
            raise ValueError("fit must be called before transform!")
        
        X_array = X.values if hasattr(X, 'values') else np.asarray(X)
        return X_array[:, self.final_indices_]


class SelectKBestPairwiseCorrMedian(BasePairwiseBacteriaSelector):
    """
    Feature selector with correlation clustering and median feature selection.

    1. Pairwise SelectKBest to collect initial features
    2. Correlation-based clustering
    3. From each cluster, select the feature with median correlation to others.
    """
    
    def __init__(self, k=20, score_func=f_classif, corr_threshold=0.8):
        """
        Initialize the selector.
        
        Args:
            k (int): Number of top features to select in each pairwise comparison
            score_func (callable): Scoring function for SelectKBest
            corr_threshold (float): Correlation threshold for clustering
        """
        super().__init__(k=k, score_func=score_func, corr_threshold=corr_threshold)
        self.feature_clusters_ = None
        self.final_indices_ = None
        self.feature_names_out_ = None

    def fit(self, X, y):
        """
        Fit the selector by clustering and selecting median feature per cluster.
        
        Args:
            X: Training features
            y: Target labels
            
        Returns:
            self: Fitted transformer
        """
        # Prepare data
        X_array, y_array, self.feature_names_in_ = self._prepare_data(X, y)
        
        # Pairwise SelectKBest
        union_selected_features, _ = self._pairwise_select_k_best(X_array, y_array)
        
        # Get selected features
        selected_feature_names = sorted(list(union_selected_features))
        initial_indices = [self.feature_names_in_.index(f) for f in selected_feature_names]
        X_selected = X_array[:, initial_indices]
        
        # Correlation clustering
        X_selected_df = pd.DataFrame(X_selected, columns=selected_feature_names)
        correlation_matrix = X_selected_df.corr().fillna(0)
        
        distance_matrix = 1 - np.abs(correlation_matrix.values)
        np.fill_diagonal(distance_matrix, 0)
        
        linkage_matrix = linkage(squareform(distance_matrix), method='complete')
        clusters = fcluster(linkage_matrix, t=1 - self.corr_threshold, criterion='distance')
        
        feature_clusters = {}
        for feature, cluster_id in zip(selected_feature_names, clusters):
            feature_clusters.setdefault(cluster_id, []).append(feature)
        self.feature_clusters_ = feature_clusters
        
        # Select median feature per cluster
        kept_features = []
        for cluster_id, features in sorted(feature_clusters.items()):
            # Get indices of features in this cluster
            cluster_indices = [selected_feature_names.index(f) for f in features]
            
            # Extract correlation matrix for this cluster
            cluster_corr_matrix = correlation_matrix.iloc[cluster_indices, cluster_indices]
            
            # Calculate mean absolute correlation for each feature to others in cluster
            mean_corr = cluster_corr_matrix.abs().mean(axis=1)
            
            # Select feature with median correlation
            median_idx = mean_corr.argsort().iloc[len(mean_corr) // 2]
            median_feature = features[median_idx]
            
            kept_features.append(median_feature)
        
        self.feature_names_out_ = kept_features
        self.final_indices_ = [self.feature_names_in_.index(f) for f in kept_features]
        
        
        return self

    def transform(self, X):
        """
        Transform X by selecting only the median features.
        
        Args:
            X: Data to transform
            
        Returns:
            ndarray: Transformed data with selected features
        """
        if self.final_indices_ is None:
            raise ValueError("Fit must be called before transform.")
        
        X_array = X.values if hasattr(X, 'values') else np.asarray(X)
        return X_array[:, self.final_indices_]


class SelectKBestPairwiseCorrVar(BasePairwiseBacteriaSelector):
    """
    Feature selector with correlation clustering and variance-based selection.
    
    1. Pairwise SelectKBest to collect initial features
    2. Correlation-based clustering
    3. From each cluster, select the feature with the highest variance
    """
    
    def __init__(self, k=20, score_func=f_classif, corr_threshold=0.8):
        """
        Initialize the selector.
        
        Args:
            k (int): Number of top features to select in each pairwise comparison
            score_func (callable): Scoring function for SelectKBest
            corr_threshold (float): Correlation threshold for clustering
        """
        super().__init__(k=k, score_func=score_func, corr_threshold=corr_threshold)
        self.feature_clusters_ = None
        self.final_indices_ = None
        self.feature_names_out_ = None

    def fit(self, X, y):
        """
        Fit the selector.

        """
        # Prepare data
        X_array, y_array, self.feature_names_in_ = self._prepare_data(X, y)
        
        # Pairwise SelectKBest
        union_selected_features, _ = self._pairwise_select_k_best(X_array, y_array)
        
        # Get selected features
        selected_feature_names = sorted(list(union_selected_features))
        initial_indices = [self.feature_names_in_.index(f) for f in selected_feature_names]
        X_selected = X_array[:, initial_indices]
        
        # Correlation clustering
        X_selected_df = pd.DataFrame(X_selected, columns=selected_feature_names)
        correlation_matrix = X_selected_df.corr().fillna(0)
        
        distance_matrix = 1 - np.abs(correlation_matrix.values)
        np.fill_diagonal(distance_matrix, 0)
        
        linkage_matrix = linkage(squareform(distance_matrix), method='complete')
        clusters = fcluster(linkage_matrix, t=1 - self.corr_threshold, criterion='distance')
        
        feature_clusters = {}
        for feature, cluster_id in zip(selected_feature_names, clusters):
            feature_clusters.setdefault(cluster_id, []).append(feature)
        self.feature_clusters_ = feature_clusters
        
        # Select feature with highest variance per cluster
        kept_features = []
        for cluster_id, features in sorted(feature_clusters.items()):
            # Calculate variance for each feature in the cluster
            variances = X_selected_df[features].var(axis=0)
            
            # Select feature with maximum variance
            best_feature = features[variances.argmax()]
            kept_features.append(best_feature)
        
        self.feature_names_out_ = kept_features
        self.final_indices_ = [self.feature_names_in_.index(f) for f in kept_features]
        
        
        return self

    def transform(self, X):
        """
        Transform X by selecting only the highest-variance features.
        
        Args:
            X: Data to transform
            
        Returns:
            ndarray: Transformed data with selected features
        """
        if self.final_indices_ is None:
            raise ValueError("Fit must be called before transform!")
        
        X_array = X.values if hasattr(X, 'values') else np.asarray(X)
        return X_array[:, self.final_indices_]


class SelectKBestPairwiseCorrMax(BasePairwiseBacteriaSelector):
    """
    Feature selector with correlation clustering and maximum correlation selection.
    
    1. Pairwise SelectKBest to collect initial features
    2. Correlation-based clustering
    3. From each cluster, select the feature with highest average correlation to others
    """
    
    def __init__(self, k=20, score_func=f_classif, corr_threshold=0.8):
        """
        Initialize the selector.
        
        Args:
            k (int): Number of top features to select in each pairwise comparison
            score_func (callable): Scoring function for SelectKBest
            corr_threshold (float): Correlation threshold for clustering
        """
        super().__init__(k=k, score_func=score_func, corr_threshold=corr_threshold)
        self.feature_clusters_ = None
        self.final_indices_ = None
        self.feature_names_out_ = None

    def fit(self, X, y):
        """
        Fit the selector.
        
        Args:
            X: Training features
            y: Target labels
            
        Returns:
            self: Fitted transformer
        """
        # Prepare data
        X_array, y_array, self.feature_names_in_ = self._prepare_data(X, y)
        
        # Pairwise SelectKBest
        union_selected_features, _ = self._pairwise_select_k_best(X_array, y_array)
        
        # Get selected features
        selected_feature_names = sorted(list(union_selected_features))
        initial_indices = [self.feature_names_in_.index(f) for f in selected_feature_names]
        X_selected = X_array[:, initial_indices]
        
        # Correlation clustering
        X_selected_df = pd.DataFrame(X_selected, columns=selected_feature_names)
        correlation_matrix = X_selected_df.corr().fillna(0)
        
        distance_matrix = 1 - np.abs(correlation_matrix.values)
        np.fill_diagonal(distance_matrix, 0)
        
        linkage_matrix = linkage(squareform(distance_matrix), method='complete')
        clusters = fcluster(linkage_matrix, t=1 - self.corr_threshold, criterion='distance')
        
        feature_clusters = {}
        for feature, cluster_id in zip(selected_feature_names, clusters):
            feature_clusters.setdefault(cluster_id, []).append(feature)
        self.feature_clusters_ = feature_clusters
        
        # Select feature with maximum average correlation per cluster
        kept_features = []
        for cluster_id, features in sorted(feature_clusters.items()):
            # Get indices of features in this cluster
            cluster_indices = [selected_feature_names.index(f) for f in features]
            
            # Extract correlation matrix for this cluster
            cluster_corr_matrix = correlation_matrix.iloc[cluster_indices, cluster_indices]
            
            # Calculate mean absolute correlation for each feature to others
            mean_corr = cluster_corr_matrix.abs().mean(axis=1)
            
            # Select feature with maximum correlation
            max_idx = mean_corr.argmax()
            max_corr_feature = features[max_idx]
            
            kept_features.append(max_corr_feature)
        
        self.feature_names_out_ = kept_features
        self.final_indices_ = [self.feature_names_in_.index(f) for f in kept_features]
        
        
        return self

    def transform(self, X):
        """
        Transform X by selecting only the max-correlation features.
        
        Args:
            X: Data to transform
            
        Returns:
            ndarray: Transformed data with selected features
        """
        if self.final_indices_ is None:
            raise ValueError("Fit must be called before transform!")
        
        X_array = X.values if hasattr(X, 'values') else np.asarray(X)
        return X_array[:, self.final_indices_]


# ====================================================================
# day-based cross validation
# ====================================================================

class TimeBasedCVSplitter:
    """
    Time-based cross-validation splitter.
    
    Creates train/test splits based on date information,
    using a 2 days training, 1 day testing strategy.
    
    Attributes:
        date_column (str): Name of the column containing date information
        splits_ (list): List of train/test split dictionaries
    """
    
    def __init__(self, date_column='Date[yyyy-mm-dd]'):
        """
        Initialize the CV splitter.
        
        Args:
            date_column (str): Name of the date column in the DataFrame
        """
        self.date_column = date_column
        self.splits_ = []
    
    def create_splits(self, df):
        """
        Create time-based splits: 2 days training, 1 day testing.
        
        This method generates all possible combinations where:
        - Training set: 2 days
        - Test set: 1 remaining day
        
        Args:
            df (DataFrame): Input dataframe with date column
            
        Returns:
            list: List of split dictionaries containing train/test indices and days
        """
        unique_days = sorted(df[self.date_column].unique())
        
        if len(unique_days) < 3:
            raise ValueError(f"At least 3 days required, but only {len(unique_days)} found")
        
        self.splits_ = []
        
        # Generate all combinations of 2 training days
        for train_days in combinations(unique_days, 2):
            remaining_days = [day for day in unique_days if day not in train_days]
            
            # Each remaining day becomes a test set
            for test_day in remaining_days:
                train_indices = df[df[self.date_column].isin(train_days)].index.tolist()
                test_indices = df[df[self.date_column] == test_day].index.tolist()
                
                # Only include splits with sufficient samples
                if len(train_indices) > 0 and len(test_indices) > 0:
                    self.splits_.append({
                        'train_indices': train_indices,
                        'test_indices': test_indices,
                        'train_days': train_days,
                        'test_day': test_day
                    })
        
        print(f"Created {len(self.splits_)} CV splits from {len(unique_days)} days")
        return self.splits_
    
    def get_n_splits(self):
        """Get the number of CV splits."""
        return len(self.splits_)
    
    def split(self, X, y=None):
        """
        Generator for sklearn-compatible cross-validation
        
        Args:
            X: Feature matrix 
            y: Target vector 
            
        Yields:
            tuple: (train_indices, test_indices) for each split
        """
        for split in self.splits_:
            yield split['train_indices'], split['test_indices']


# ====================================================================
# MODEL EVALUATION
# ====================================================================

def comprehensive_model_evaluation(features_df, date_column='Date[yyyy-mm-dd]', target_column='Class'):
    """
    Model evaluation with GridSearchCV and time-based cross-validation.
    
    This function:
    1. Prepares the dataset
    2. Creates time-based CV splits
    3. Defines multiple model variants (different feature selectors × classifiers)
    4. Performs GridSearchCV for hyperparameter tuning
    5. Evaluates each model on all CV folds
    6. Extracts feature importance
    7. Compares all models and ranks them by performance
    
    Args:
        features_df (DataFrame): Input dataframe with features, labels, and dates
        date_column (str): Name of the date column
        target_column (str): Name of the target/class column
        
    Returns:
        tuple: (all_results, comparison) where:
            - all_results is a dict mapping model names to detailed results
            - comparison is a list of model performance comparisons
    """
    
    # Prepare data
    df = features_df.copy()
    df = df.reset_index(drop=True)  
    
    # Separate features and labels
    feature_columns = [col for col in df.columns if col not in [date_column, target_column]]
    X = df[feature_columns] 
    y = df[target_column].values  
    # Store feature names for later use
    original_feature_names = feature_columns
    
    print(f"Dataset: {X.shape[0]} samples, {X.shape[1]} features, {len(np.unique(y))} classes")
    print(f"Classes: {sorted(np.unique(y))}")
    
    # Create CV splitter
    cv_splitter = TimeBasedCVSplitter(date_column)
    cv_splits = cv_splitter.create_splits(df)
    
    # ====================================================================
    # MODEL DEFINITIONS
    # ====================================================================
    
    models_config = {}
    
    def add_model_variants(base_name, classifier, scaler, classifier_params):
        """
        Add multiple feature selection variants for a given classifier.
        Args:
            base_name (str): Base name for the model (e.g., 'SVM_Standard')
            classifier: Sklearn classifier instance
            scaler: Sklearn scaler instance
            classifier_params (dict): Grid search parameters for the classifier
        """
        # Variant 1: BacteriaFeatureSelector - simple union of pairwise features
        models_config[f'{base_name}_BactSelect'] = {
            'pipeline': Pipeline([
                ('feature_selector', BacteriaFeatureSelector(k=10)),
                ('scaler', scaler),
                ('classifier', classifier)
            ]),
            'param_grid': {
                'feature_selector__k': [15, 20, 25],
                **{f'classifier__{k}': v for k, v in classifier_params.items()}
            }
        }
        
        # Variant 2: Correlation Aggregator - clusters and averages correlated features
        models_config[f'{base_name}_CorrAgg'] = {
            'pipeline': Pipeline([
                ('feature_selector', SelectKBestPairwiseCorrAggregator(k=20, corr_threshold=0.5)),
                ('scaler', scaler),
                ('classifier', classifier)
            ]),
            'param_grid': {
                'feature_selector__k': [15, 20, 25],
                'feature_selector__corr_threshold': [0.7, 0.8, 0.9],
                **{f'classifier__{k}': v for k, v in classifier_params.items()}
            }
        }
        
        # Variant 3: Correlation Dropper - keeps first feature from each cluster
        models_config[f'{base_name}_CorrDrop'] = {
            'pipeline': Pipeline([
                ('feature_selector', SelectKBestPairwiseCorrDropper(k=20, corr_threshold=0.5)),
                ('scaler', scaler),
                ('classifier', classifier)
            ]),
            'param_grid': {
                'feature_selector__k': [15, 20, 25],
                'feature_selector__corr_threshold': [0.7, 0.8, 0.9],
                **{f'classifier__{k}': v for k, v in classifier_params.items()}
            }
        }
        
        # Variant 4: Correlation + LDA - applies LDA to each cluster
        models_config[f'{base_name}_CorrLDA'] = {
            'pipeline': Pipeline([
                ('feature_selector', SelectKBestCorrelationLDA(k=20, corr_threshold=0.9, max_lda_components=3)),
                ('scaler', scaler),
                ('classifier', classifier)
            ]),
            'param_grid': {
                'feature_selector__k': [15, 20, 25],
                'feature_selector__corr_threshold': [0.7, 0.8, 0.9],
                'feature_selector__max_lda_components': [2, 3, 4, 5],
                **{f'classifier__{k}': v for k, v in classifier_params.items()}
            }
        }
        
        # Variant 5: Pairwise + Correlation + Best Score - keeps highest-scoring feature
        models_config[f'{base_name}_PairwiseCorrBest'] = {
            'pipeline': Pipeline([
                ('feature_selector', SelectKBestPairwiseCorrBestScore(k=20, corr_threshold=0.9)),
                ('scaler', scaler),
                ('classifier', classifier)
            ]),
            'param_grid': {
                'feature_selector__k': [15, 20, 25],
                'feature_selector__corr_threshold': [0.7, 0.8, 0.9],
                **{f'classifier__{k}': v for k, v in classifier_params.items()}
            }
        }
        
        # Variant 7: Correlation + Median - keeps median-correlation feature
        models_config[f'{base_name}_CorrMedian'] = {
            'pipeline': Pipeline([
                ('feature_selector', SelectKBestPairwiseCorrMedian(k=10, corr_threshold=0.8)),
                ('scaler', scaler),
                ('classifier', classifier)
            ]),
            'param_grid': {
                'feature_selector__k': [15, 20, 25],
                'feature_selector__corr_threshold': [0.7, 0.8, 0.9],
                **{f'classifier__{k}': v for k, v in classifier_params.items()}
            }
        }
        
        # Variant 8: Correlation + Max Variance - keeps highest-variance feature
        models_config[f'{base_name}_CorrVar'] = {
            'pipeline': Pipeline([
                ('feature_selector', SelectKBestPairwiseCorrVar(k=10, corr_threshold=0.8)),
                ('scaler', scaler),
                ('classifier', classifier)
            ]),
            'param_grid': {
                'feature_selector__k': [15, 20, 25],
                'feature_selector__corr_threshold':[ 0.7, 0.8, 0.9],
                **{f'classifier__{k}': v for k, v in classifier_params.items()}
            }
        }
        
        
        # Variant 9: Correlation + Max Correlation - keeps most correlated feature
        models_config[f'{base_name}_CorrMax'] = {
            'pipeline': Pipeline([
                ('feature_selector', SelectKBestPairwiseCorrMax(k=10, corr_threshold=0.8)),
                ('scaler', scaler),
                ('classifier', classifier)
            ]),
            'param_grid': {
                'feature_selector__k': [15, 20, 25],
                'feature_selector__corr_threshold': [0.7, 0.8, 0.9],
                **{f'classifier__{k}': v for k, v in classifier_params.items()}
            }
        }
    
    # Add all classifier × feature selector combinations
    
    # Logistic Regression with Standard Scaler
    add_model_variants(
        'LogReg_Standard',
        LogisticRegression(random_state=42, max_iter=1000),
        StandardScaler(),
        {
            'C': [0.01, 0.1, 1.0, 10.0],
            'penalty': ['l2'],
            'solver': ['lbfgs']
        }
    )
    
    # Logistic Regression with Robust Scaler
    add_model_variants(
        'LogReg_Robust',
        LogisticRegression(random_state=42, max_iter=1000),
        RobustScaler(),
        {
            'C': [0.01, 0.1, 1.0, 10.0],
            'penalty': ['l2'],
            'solver': ['lbfgs']
        }
    )
    
    # SVM with Standard Scaler
    add_model_variants(
        'SVM_Standard',
        SVC(random_state=42, probability=False),
        StandardScaler(),
        {
            'C': [0.1, 1.0, 10.0],
            'kernel': ['linear', 'rbf'],
            'gamma': ['scale']
        }
    )
    
    # SVM with Robust Scaler
    add_model_variants(
        'SVM_Robust',
        SVC(random_state=42, probability=False),
        RobustScaler(),
        {
            'C': [0.1, 1.0, 10.0],
            'kernel': ['linear', 'rbf'],
            'gamma': ['scale']
        }
    )
    
    # Random Forest 
    add_model_variants(
        'RandomForest',
        RandomForestClassifier(random_state=42),
        StandardScaler(),
        {
            'n_estimators': [50, 100, 200],
            'max_depth': [5, 10, None],
            'min_samples_split': [2, 5]
        }
    )
    
    # Gradient Boosting with Robust Scaler
    add_model_variants(
        'GradientBoosting_Robust',
        GradientBoostingClassifier(random_state=42),
        RobustScaler(),
        {
            'n_estimators': [50, 100],
            'learning_rate': [0.01, 0.1, 0.2],
            'max_depth': [3, 5]
        }
    )
    
    # Gradient Boosting with Standard Scaler
    add_model_variants(
        'GradientBoosting_Standard',
        GradientBoostingClassifier(random_state=42),
        StandardScaler(),
        {
            'n_estimators': [50, 100],
            'learning_rate': [0.01, 0.1, 0.2],
            'max_depth': [3, 5]
        }
    )
    
    # k-Nearest Neighbors
    add_model_variants(
        'kNN',
        KNeighborsClassifier(),
        StandardScaler(),
        {
            'n_neighbors': [3, 5, 7, 9, 11, 13, 15],
            'weights': ['uniform', 'distance'],
            'metric': ['euclidean', 'manhattan']
        }
    )
    
    # Decision Tree with Standard Scaler
    add_model_variants(
        'DecisionTree_Standard',
        DecisionTreeClassifier(random_state=42),
        StandardScaler(),
        {
            'max_depth': [3, 5, 7, 10, None],
            'min_samples_split': [2, 5, 10, 20],
            'min_samples_leaf': [1, 5, 10],
            'criterion': ['gini', 'entropy']
        }
    )
    
    # Decision Tree with Robust Scaler
    add_model_variants(
        'DecisionTree_Robust',
        DecisionTreeClassifier(random_state=42),
        RobustScaler(),
        {
            'max_depth': [3, 5, 7, 10, None],
            'min_samples_split': [2, 5, 10, 20],
            'min_samples_leaf': [1, 5, 10],
            'criterion': ['gini', 'entropy']
        }
    )
    
    print(f"\nCreated {len(models_config)} model variants:")
    for name in sorted(models_config.keys()):
        print(f"  - {name}")
    
    # ====================================================================
    # EVALUATION OF ALL MODELS WITH GRIDSEARCHCV
    # ====================================================================
    
    all_results = {}
    
    for model_name, config in models_config.items():
        print(f"\n{'='*80}")
        print(f"EVALUATING MODEL: {model_name}")
        print(f"{'='*80}")
        
        pipeline = config['pipeline']
        param_grid = config['param_grid']
        
        # GridSearchCV 
        grid_search = GridSearchCV(
            estimator=pipeline,
            param_grid=param_grid,
            cv=list(cv_splitter.split(X, y)),  
            scoring='accuracy',
            n_jobs=-1,  
            verbose=1,
            return_train_score=True,
            error_score='raise'
        )
        
        try:
            # Encoding labels
            le = LabelEncoder()
            y_encoded = le.fit_transform(y)

            
            # Fit Grid Search to find best hyperparameters
            grid_search.fit(X, y_encoded)
            
            # Extract best parameters and scores
            best_params = grid_search.best_params_
            best_score = grid_search.best_score_
            
            print(f"\nBEST PARAMETERS:")
            for param, value in best_params.items():
                print(f"  {param}: {value}")
            print(f"\nBest CV Accuracy: {best_score:.4f}")
            
            # Evaluate on each CV fold with best parameters
            fold_results = []
            all_y_true = []
            all_y_pred = []
            
            for split in cv_splits:
                train_idx = split['train_indices']
                test_idx = split['test_indices']
                
                X_train, X_test = X.iloc[train_idx], X.iloc[test_idx]
                y_train, y_test = y_encoded[train_idx], y_encoded[test_idx]
                
                # Train model on this fold
                model_clone = clone(grid_search.best_estimator_)
                model_clone.fit(X_train, y_train)
                y_pred = model_clone.predict(X_test)
                
                accuracy = accuracy_score(y_test, y_pred)
                
                fold_results.append({
                    'train_days': split['train_days'],
                    'test_day': split['test_day'],
                    'accuracy': accuracy,
                    'n_samples': len(y_test)
                })
                
                all_y_true.extend(y_test)
                all_y_pred.extend(y_pred)
            
            # Calculate statistics across folds
            accuracies = [r['accuracy'] for r in fold_results]
            print(f"\nFOLD STATISTICS:")
            print(f"Mean Accuracy: {np.mean(accuracies):.4f} ± {np.std(accuracies):.4f}")
            print(f"Min/Max: {np.min(accuracies):.4f} / {np.max(accuracies):.4f}")
            fold_details = ", ".join([f"Fold {i+1}: {acc:.4f}" for i, acc in enumerate(accuracies)])
            print(f"Individual Folds: {fold_details}")
            
            # Classification Report (aggregated across all folds)
            all_y_true_decoded = le.inverse_transform(all_y_true)
            all_y_pred_decoded = le.inverse_transform(all_y_pred)
            
        
            print(f"\nCLASSIFICATION REPORT:")
            print(classification_report(all_y_true_decoded, all_y_pred_decoded, zero_division=0))
            
            # Confusion Matrix (aggregated across all folds)
            print(f"\nCONFUSION MATRIX:")
            cm = confusion_matrix(all_y_true_decoded, all_y_pred_decoded)
            
            classes = le.classes_ 
            cm_df = pd.DataFrame(cm, index=classes, columns=classes)
            print(cm_df)
            
            # Save all results for this model
            all_results[model_name] = {
                'best_params': best_params,
                'best_score': best_score,
                'cv_results': grid_search.cv_results_,
                'fold_results': fold_results,
                'mean_accuracy': np.mean(accuracies),
                'std_accuracy': np.std(accuracies),
                'all_y_true': all_y_true,
                'all_y_pred': all_y_pred,
            }
            
        except Exception as e:
            print(f"ERROR in {model_name}: {e}")
            import traceback
            traceback.print_exc()
            continue
    
    # ====================================================================
    # FINAL MODEL COMPARISON
    # ====================================================================
    
    print(f"\n{'='*80}")
    print(f"FINAL MODEL COMPARISON")
    print(f"{'='*80}\n")
    
    comparison = []
    for model_name, results in all_results.items():
        comparison.append({
            'Model': model_name,
            'CV_Score': results['best_score'],
            'Mean_Accuracy': results['mean_accuracy'],
            'Std_Accuracy': results['std_accuracy'],
            'Best_Params': results['best_params']
        })
    
    # Sort by CV score (descending)
    comparison.sort(key=lambda x: x['CV_Score'], reverse=True)
    
    # Print comparison table
    print(f"{'Rank':<5} {'Model':<25} {'CV Score':<12} {'Mean Acc':<12} {'Std':<10}")
    print("-" * 80)
    for i, result in enumerate(comparison):
        print(f"{i+1:<5} {result['Model']:<25} {result['CV_Score']:<12.4f} "
              f"{result['Mean_Accuracy']:<12.4f} {result['Std_Accuracy']:<10.4f}")
    
    # Print winner
    if comparison:
        print(f"\nWINNER: {comparison[0]['Model']}")
        print(f"CV Score: {comparison[0]['CV_Score']:.4f}")
    
    return all_results, comparison


# ====================================================================
# Execute the pipeline
# ====================================================================

def run_evaluation(features_df):
    results, comparison = comprehensive_model_evaluation(
        features_df,
        date_column='Date[yyyy-mm-dd]',
        target_column='Class'
    )
    
    return results, comparison


# Run the evaluation pipeline
results, comparison = run_evaluation(features_df)
```
