# Supplementary material for: Data-driven exploration of electronic nose technology to differentiate bacteria in blood cultures under biofilm-promoting conditions
Source: Sci Rep. 2026 Jul 10;16:21641. doi: 10.1038/s41598-026-62071-8 (PMC13354572; doi:10.1038/s41598-026-62071-8)
Supplement: Supplementary file 1 — Supplementary Material 1 [file 41598_2026_62071_MOESM1_ESM.zip › Supplementary Notes.pdf]

Overview of the derived hyperparameters and pre-processing methods

Support Vector Machine (SVM)

- **All Classes:** C=1.0, γ=scale, kernel=linear; **Feature selection via:** SelectkBest (k=15); **Scaler:** RobustScaler.
- **3 Classes:** C=10.0, γ=scale, kernel=linear; **Feature selection via:** SelectKBest (k=25) + Clustering (clustering threshold=0.8) + Median fclassif score; **Scaler:** RobustScaler.
- **Biofilm vs. Control:** C=1.0, γ=scale, kernel=rbf; **Feature selection via:** SelectKBest (k=20) + Clustering (clustering threshold=0.7) + Highest fclassif score; **Scaler:** StandardScaler / RobustScaler.
- **Biofilm vs. Control (without *E. faecalis*):** C=1.0, γ=scale, kernel=linear; **Feature selection via:** SelectKBest (k=25) + Clustering (clustering threshold=0.9) + LDA (max LDA=2); **Scaler:** StandardScaler.

k-Nearest Neighbors (k-NN)

- **All Classes:** metric=euclidean, nneighbors=13, weights=distance; **Feature selection via:** SelectKBest (k=25) + Clustering (clustering threshold=0.9) + Highest fclassif score.
- **3 Classes:** metric=euclidean, nneighbors=9, weights=uniform; **Feature selection via:** SelectKBest (k=15).
- **Biofilm vs. Control:** metric=manhattan, nneighbors=5, weights=uniform; **Feature selection via:** SelectKBest (k=20) + Clustering (clustering threshold=0.9) + Highest fclassif score.
- **Biofilm vs. Control (without *E. faecalis*):** metric=manhattan, nneighbors=5, weights=uniform; **Feature selection via:** SelectKBest (k=20) + Clustering (clustering threshold=0.9) + LDA (max LDA=2).

Decision Tree

- **All Classes:** criterion=entropy, max_depth=5, min_samples_leaf=1, min_samples_split=2; **Feature selection via:** SelectKBest (k=25) + Clustering (clustering threshold=0.7) + Most correlated feature; **Scaler:** StandardScaler/RobustScaler.

- **3 Classes:** criterion=gini, max_depth=3, min_samples_leaf=1, min_samples_split=2; **Feature selection via:** SelectKBest (k=20) + Clustering (clustering threshold=0.7) + Mean of cluster features; **Scaler:** RobustScaler.
- **Biofilm vs. Control:** criterion=gini, max_depth=3, min_samples_leaf=10, min_samples_split=2; **Feature selection via:** SelectKBest (k=15) + Clustering (clustering threshold=0.8) + LDA (max LDA=2); **Scaler:** StandardScaler/RobustScaler.
- **Biofilm vs. Control (without *E. faecalis*):** criterion=gini, max_depth=3, min_samples_leaf=5, min_samples_split=2; **Feature selection via:** SelectKBest (k=15); **Scaler:** StandardScaler.

Logistic Regression

- **All Classes:** C=1.0, penalty=l2, solver=lbfgs; **Feature selection via:** SelectKBest (k=20) + Clustering (clustering threshold=0.9) + LDA (max LDA=3; **Scaler:** StandardScaler.
- **3 Classes:** C=0.01, penalty=l2, solver=lbfgs; **Feature selection via:** SelectKBest (k=15); **Scaler:** StandardScaler.
- **Biofilm vs. Control:** C=10.0, penalty=l2, solver=lbfgs; **Feature selection via:** SelectKBest (k=15) + Clustering (clustering threshold=0.7) + Highest fclassif score; **Scaler:** StandardScaler.
- **Biofilm vs. Control (without *E. faecalis*):** C=1.0, penalty=l2, solver=lbfgs; **Feature selection via:** SelectKBest (k=25) + Clustering (clustering threshold=0.9) + LDA (max LDA=2); **Scaler:** StandardScaler.

Gradient Boosting

- **All Classes:** learning_rate=0.01, max_depth=3, n_estimators=100; **Feature selection via:** SelectKBest (k=15) + Clustering (clustering threshold=0.9) + First feature of each cluster; **Scaler:** RobustScaler.
- **3 Classes:** learning_rate=0.2, max_depth=5, n_estimators=100; **Feature selection via:** SelectKBest (k=15) + Clustering (clustering threshold=0.7) + Mean of cluster features; **Scaler:** RobustScaler.
- **Biofilm vs. Control:** learning_rate=0.1, max_depth=5, n_estimators=50; **Feature selection via:** SelectKBest (k=15) + Clustering (clustering threshold=0.8) + Median fclassif score; **Scaler:** RobustScaler.

- **Biofilm vs. Control (without *E. faecalis*):** learning_rate=0.2, max_depth=5, n_estimators=50; **Feature selection via:** SelectKBest (k=25) + Clustering (clustering threshold=0.9) + LDA (max LDA=2); **Scaler:** RobustScaler.

Random Forest

- **All Classes:** max_depth=5, min_samples_split=2, n_estimators=50; **Feature selection via:** SelectKBest (k=25) + Clustering (clustering threshold=0.8) + LDA (max LDA=2).
- **3 Classes:** max_depth=5, min_samples_split=2, n_estimators=50; **Feature selection via:** SelectKBest (k=20) + Clustering (clustering threshold=0.8) + Mean of cluster features.
- **Biofilm vs. Control:** max_depth=5, min_samples_split=5, n_estimators=50; **Feature selection via:** SelectKBest (k=20) + Clustering (clustering threshold=0.9) + LDA (max LDA=2).
- **Biofilm vs. Control (without *E. faecalis*):** max_depth=5, min_samples_split=2, n_estimators=50; **Feature selection via:** SelectKBest (k=25) + Clustering (clustering threshold=0.9) + LDA (max LDA=2).
