# Supplementary material for: Data-driven exploration of electronic nose technology to differentiate bacteria in blood cultures under biofilm-promoting conditions
Source: Sci Rep. 2026 Jul 10;16:21641. doi: 10.1038/s41598-026-62071-8 (PMC13354572; doi:10.1038/s41598-026-62071-8)
Supplement: Supplementary file 1 — Supplementary Material 1 [file 41598_2026_62071_MOESM1_ESM.zip › Supplementary Tables.pdf]

Supplementary Tables

Feature Name	Description
Mean	Mean of last (10) values during sample exposure phase.
Final response	Difference of the sensor response values during sample air exposure (averaged over 10s) to recovery sensor values after sample air exposure (averaged over 10s) relative to recovery sensor values.<br>Indicates if the sensor recovers well after sample exposure.
Thirty second on response	The difference between the sensor value mean (averaged over 5s) after 30s of sample gas exposure and the baseline.<br>Indicates how quickly the sensor reacts.
Thirty second off response	The difference between the sensor value (averaged over 5s) after 30s of sample gas turned off and the baseline.<br>Indicates how quickly the sensor recovers.
Ninety second on response	The difference between the sensor value (averaged over 5s) after 90s of sample gas exposure and the baseline.<br>Indicates 'mid-term' response magnitude.
Ninety second off response	The difference between the sensor value (averaged over 5s) after 90s of sample gas turned off and the baseline.<br>Indicates 'mid-term' recovery magnitude.
Min derivative	Largest negative rate of change during whole measurement.

Max derivative	Largest positive rate of change during whole measurement.
On derivative	The average rate of change from baseline (averaged over 10s) to the sensor value 10 seconds after gas exposure starts.
Off derivative	The average rate of change from baseline (averaged over 10s) to the sensor value 10 seconds after gas exposure stops.
Plateau derivative	The mean slope from 90s after gas exposure to the end of gas exposure.<br>Measures signal stability and saturation.
On integral	The area under the response curve during sample air exposure.
Off integral	The area under recovery curve after sample air exposure.
Short off integral	The area under the recovery curve within the first 60s after sample air exposure.<br>Showing immediate desorption behavior.
Response divided by on integral	Ratio of peak response value to total response area.
Skewness	The skewness of the distribution of the measured values during sample air stage and recovery stage.<br>Metric for balance between reaction to sample air and recovery of the sensor.
Sample CV	Coefficient of variation at the end of the sample exposure stage, defined by $\frac{s}{\bar{x}}$ (calculated with 30 values, respectively).

T50 recovery	The time required to reach 50% of the final value of the recovery stage. This metric assumes a plateau at the end of the recovery stage.
Difference	The difference between sample exposure values (averaged over 10s) and baseline values (averaged over 10s).
Fractional difference	The fractional difference of baseline values (averaged over 10s) and measured resistance after sample exposure (averaged over last 10s).
Log difference	Logarithmic ratio of sample air exposure values (averaged over 10s) to the baseline values (averaged over 10s).
Relative difference	Ratio of sample air exposure values (averaged over 10s) to the baseline values (averaged over 10s).
Recovery level	Ratio of baseline values (averaged over 10s) to the sensor recovery values after sample air exposure (averaged over 10s).
Sample startslope	The rate of change in the first 30 seconds of exposure. Measures how quickly the sensor responds.
Sample endslope	The rate of change in the last 30 seconds of gas exposure.
Recovery slope	Measures how quickly the sensor recovers in the first 30 seconds after sample gas is turned off.

Table S1: Overview of all extracted features their definitions.

	Control	E. faecalis	P. aeruginosa	S. aureus	S. epidermidis
**Control**	**7**	3	0	2	0
**E. faecalis**	4	**5**	0	2	1
**P. aeruginosa**	0	0	**11**	0	0
**S. aureus**	1	1	1	**7**	1
**S. epidermidis**	0	1	0	6	**3**

Table S2: Confusion matrix of the Gradient Boosting model for the cross validation and five-class-problem.

	Control	E. faecalis	P. aeruginosa	S. aureus	S. epidermidis
**Control**	**1**	5	0	5	1
**E. faecalis**	2	**8**	0	2	0
**P. aeruginosa**	0	0	**11**	0	0
**S. aureus**	3	1	0	**6**	1
**S. epidermidis**	0	1	0	6	**3**

Table S3: Confusion matrix of the kNN model for the cross validation and five-class-problem.

	Control	P. aeruginosa	S. epidermidis
**Control**	**11**	0	1
**P. aeruginosa**	0	**11**	0
**S. epidermidis**	1	1	**8**

Table S4: Confusion matrix of the Random Forest classifier for the cross validation and three-class-problem.

	Control	P. aeruginosa	S. epidermidis
**Control**	**12**	0	0
**P. aeruginosa**	0	**11**	0
**S. epidermidis**	4	0	**6**

Table S5: Confusion matrix of the kNN classifier for the cross validation and three-class-problem.

Feature	Comparison	Mean_Difference	Hedges_g	p_value	p_corrected
mean_R33[Ohm]	Sepi vs Pae	-99875.0918	-1.4271	0.0030	0.0045
mean_R33[Ohm]	Sepi vs Kontrolle	105253.5900	2.6143	0.0000	0.0000
mean_R33[Ohm]	Pae vs Kontrolle	205128.6818	2.9864	0.0000	0.0000
mean_R34[Ohm]	Sepi vs Pae	-95581.0291	-1.3913	0.0048	0.0070
mean_R34[Ohm]	Sepi vs Kontrolle	103000.6300	2.4657	0.0002	0.0003
mean_R34[Ohm]	Pae vs Kontrolle	198581.6591	3.0434	0.0000	0.0000
final_response_R3	Sepi vs Pae	-0.1113	-2.2844	0.0002	0.0003
final_response_R3	Sepi vs Kontrolle	0.0618	1.1575	0.0100	0.0143
final_response_R3	Pae vs Kontrolle	0.1731	4.5260	0.0000	0.0000
final_response_R33	Sepi vs Pae	-0.1382	-2.8961	0.0000	0.0000
final_response_R33	Sepi vs Kontrolle	0.0876	1.8934	0.0002	0.0003
final_response_R33	Pae vs Kontrolle	0.2258	7.7549	0.0000	0.0000
final_response_R34	Sepi vs Pae	-0.1255	-2.5685	0.0000	0.0000
final_response_R34	Sepi vs Kontrolle	0.0860	1.8362	0.0000	0.0000
final_response_R34	Pae vs Kontrolle	0.2115	7.9159	0.0000	0.0000
final_response_R45	Sepi vs Pae	-0.0842	-1.5731	0.0022	0.0033
final_response_R45	Sepi vs Kontrolle	0.0838	1.5417	0.0016	0.0024
final_response_R45	Pae vs Kontrolle	0.1680	3.6102	0.0000	0.0000
thirty_s_off_resp_R33	Sepi vs Pae	-19550.4264	-0.4679	0.2738	0.3476
thirty_s_off_resp_R33	Sepi vs Kontrolle	61497.0267	1.5916	0.0008	0.0012
thirty_s_off_resp_R33	Pae vs Kontrolle	81047.4530	1.9546	0.0000	0.0000
thirty_s_off_resp_R34	Sepi vs Pae	-17934.4618	-0.4300	0.3180	0.3921
thirty_s_off_resp_R34	Sepi vs Kontrolle	54542.0783	1.9478	0.0000	0.0000
thirty_s_off_resp_R34	Pae vs Kontrolle	72476.5402	1.8593	0.0006	0.0010
ninety_s_on_resp_R34	Sepi vs Pae	-242911.8255	-3.6726	0.0000	0.0000
ninety_s_on_resp_R34	Sepi vs Kontrolle	42084.5450	0.8253	0.0488	0.0659
ninety_s_on_resp_R34	Pae vs Kontrolle	284996.3705	6.0587	0.0000	0.0000
ninety_s_off_resp_R34	Sepi vs Pae	4290.6182	0.1188	0.7802	0.8790
ninety_s_off_resp_R34	Sepi vs Kontrolle	31348.1750	1.6069	0.0010	0.0015
ninety_s_off_resp_R34	Pae vs Kontrolle	27057.5568	0.7358	0.0892	0.1181
min_deriv_R49	Sepi vs Pae	12903.9727	0.3559	0.3940	0.4814
min_deriv_R49	Sepi vs Kontrolle	-34033.4667	-0.8902	0.0424	0.0578
min_deriv_R49	Pae vs Kontrolle	-46937.4394	-1.1826	0.0088	0.0127
max_deriv_R36	Sepi vs Pae	-57470.3364	-1.8602	0.0000	0.0000

max_deriv_R36	Sepi vs Kontrolle	8350.2167	1.2265	0.0076	0.0110
max_deriv_R36	Pae vs Kontrolle	65820.5530	2.2470	0.0000	0.0000
max_deriv_R38	Sepi vs Pae	-134792.2182	-2.3434	0.0000	0.0000
max_deriv_R38	Sepi vs Kontrolle	-17354.4000	-0.9816	0.0252	0.0345
max_deriv_R38	Pae vs Kontrolle	117437.8182	2.1469	0.0000	0.0000
on_derivative_R29	Sepi vs Pae	1604.1723	0.4999	0.2742	0.3476
on_derivative_R29	Sepi vs Kontrolle	-403.1900	-0.1280	0.7996	0.8864
on_derivative_R29	Pae vs Kontrolle	-2007.3623	-1.0475	0.0196	0.0273
on_derivative_R32	Sepi vs Pae	254.7077	0.1244	0.8260	0.9051
on_derivative_R32	Sepi vs Kontrolle	-482.9500	-0.2371	0.6158	0.7229
on_derivative_R32	Pae vs Kontrolle	-737.6577	-0.5196	0.2080	0.2700
off_derivative_R33	Sepi vs Pae	-4221.6941	-0.7046	0.1050	0.1383
off_derivative_R33	Sepi vs Kontrolle	9253.3183	2.0514	0.0000	0.0000
off_derivative_R33	Pae vs Kontrolle	13475.0124	2.3720	0.0000	0.0000
off_derivative_R34	Sepi vs Pae	-4330.0856	-0.8780	0.0550	0.0739
off_derivative_R34	Sepi vs Kontrolle	7798.9188	2.1414	0.0000	0.0000
off_derivative_R34	Pae vs Kontrolle	12129.0045	2.9130	0.0000	0.0000
on_integral_R34	Sepi vs Pae	-29835477.7300	-2.9686	0.0000	0.0000
on_integral_R34	Sepi vs Kontrolle	11303327.7200	1.2995	0.0014	0.0021
on_integral_R34	Pae vs Kontrolle	41138805.4500	6.6272	0.0000	0.0000
short_off_integral_R33	Sepi vs Pae	-1354797.7136	-0.7676	0.0828	0.1101
short_off_integral_R33	Sepi vs Kontrolle	3009296.2250	1.6784	0.0010	0.0015
short_off_integral_R33	Pae vs Kontrolle	4364093.9386	2.8380	0.0000	0.0000
short_off_integral_R34	Sepi vs Pae	-1431572.5600	-0.7929	0.0746	0.0997
short_off_integral_R34	Sepi vs Kontrolle	2573281.4983	1.7543	0.0008	0.0012
short_off_integral_R34	Pae vs Kontrolle	4004854.0583	2.5342	0.0000	0.0000
response_on_integral_R34	Sepi vs Pae	0.0336	1.6890	0.0000	0.0000
response_on_integral_R34	Sepi vs Kontrolle	-0.0891	-0.6098	0.0216	0.0299
response_on_integral_R34	Pae vs Kontrolle	-0.1227	-0.8700	0.0000	0.0000
skewness_R7	Sepi vs Pae	-2.1634	-5.0496	0.0002	0.0003
skewness_R7	Sepi vs Kontrolle	0.1131	0.3385	0.5024	0.6056
skewness_R7	Pae vs Kontrolle	2.2765	7.0368	0.0000	0.0000
skewness_R8	Sepi vs Pae	-2.2211	-5.5555	0.0000	0.0000
skewness_R8	Sepi vs Kontrolle	0.0425	0.1421	0.7668	0.8699
skewness_R8	Pae vs Kontrolle	2.2636	6.9495	0.0000	0.0000
skewness_R11	Sepi vs Pae	-2.3045	-4.8934	0.0000	0.0000
skewness_R11	Sepi vs Kontrolle	-0.0920	-0.2361	0.5888	0.6973
skewness_R11	Pae vs Kontrolle	2.2125	8.1131	0.0000	0.0000
skewness_R12	Sepi vs Pae	-2.1513	-4.6818	0.0000	0.0000
skewness_R12	Sepi vs Kontrolle	0.0033	0.0089	0.9832	0.9854
skewness_R12	Pae vs Kontrolle	2.1546	7.7423	0.0000	0.0000
skewness_R13	Sepi vs Pae	-1.9896	-3.4339	0.0000	0.0000
skewness_R13	Sepi vs Kontrolle	0.1039	0.2046	0.6956	0.8026
skewness_R13	Pae vs Kontrolle	2.0934	6.8192	0.0000	0.0000
skewness_R14	Sepi vs Pae	-2.0043	-3.8702	0.0000	0.0000
skewness_R14	Sepi vs Kontrolle	-0.0201	-0.0447	0.9324	0.9683
skewness_R14	Pae vs Kontrolle	1.9842	6.2127	0.0000	0.0000
skewness_R15	Sepi vs Pae	-2.1972	-5.0794	0.0000	0.0000
skewness_R15	Sepi vs Kontrolle	-0.0972	-0.3559	0.4594	0.5562

skewness_R15	Pae vs Kontrolle	2.1000	5.8420	0.0000	0.0000
skewness_R17	Sepi vs Pae	-1.4821	-2.5609	0.0002	0.0003
skewness_R17	Sepi vs Kontrolle	0.4361	0.8259	0.0432	0.0586
skewness_R17	Pae vs Kontrolle	1.9183	5.4311	0.0000	0.0000
skewness_R23	Sepi vs Pae	-1.5374	-2.5767	0.0000	0.0000
skewness_R23	Sepi vs Kontrolle	0.2834	0.5211	0.2324	0.3002
skewness_R23	Pae vs Kontrolle	1.8208	6.2552	0.0000	0.0000
skewness_R24	Sepi vs Pae	-1.5511	-2.3557	0.0000	0.0000
skewness_R24	Sepi vs Kontrolle	0.2556	0.4315	0.3526	0.4327
skewness_R24	Pae vs Kontrolle	1.8067	6.9937	0.0000	0.0000
skewness_R34	Sepi vs Pae	-0.3386	-1.7472	0.0008	0.0012
skewness_R34	Sepi vs Kontrolle	-0.2142	-0.4432	0.2974	0.3717
skewness_R34	Pae vs Kontrolle	0.1244	0.2601	0.5396	0.6447
skewness_R41	Sepi vs Pae	-1.4998	-2.2695	0.0000	0.0000
skewness_R41	Sepi vs Kontrolle	0.1725	0.2871	0.5204	0.6245
skewness_R41	Pae vs Kontrolle	1.6723	6.3652	0.0000	0.0000
skewness_R42	Sepi vs Pae	-1.4429	-2.3223	0.0000	0.0000
skewness_R42	Sepi vs Kontrolle	0.3220	0.5612	0.2000	0.2609
skewness_R42	Pae vs Kontrolle	1.7649	6.1075	0.0000	0.0000
skewness_R44	Sepi vs Pae	-1.4942	-2.2631	0.0000	0.0000
skewness_R44	Sepi vs Kontrolle	0.2808	0.4627	0.2800	0.3533
skewness_R44	Pae vs Kontrolle	1.7749	6.6627	0.0000	0.0000
skewness_R45	Sepi vs Pae	-1.5080	-2.2908	0.0000	0.0000
skewness_R45	Sepi vs Kontrolle	0.3030	0.4946	0.2648	0.3388
skewness_R45	Pae vs Kontrolle	1.8109	6.2050	0.0000	0.0000
skewness_R46	Sepi vs Pae	-1.4939	-2.2591	0.0002	0.0003
skewness_R46	Sepi vs Kontrolle	0.2723	0.4535	0.3156	0.3909
skewness_R46	Pae vs Kontrolle	1.7662	6.5067	0.0000	0.0000
skewness_R54	Sepi vs Pae	-1.4543	-2.2141	0.0000	0.0000
skewness_R54	Sepi vs Kontrolle	0.2258	0.3780	0.4190	0.5096
skewness_R54	Pae vs Kontrolle	1.6801	5.9196	0.0000	0.0000
skewness_R55	Sepi vs Pae	-1.4075	-2.1485	0.0002	0.0003
skewness_R55	Sepi vs Kontrolle	0.3741	0.6204	0.1444	0.1893
skewness_R55	Pae vs Kontrolle	1.7816	6.3775	0.0000	0.0000
skewness_R57	Sepi vs Pae	-1.4346	-2.1887	0.0002	0.0003
skewness_R57	Sepi vs Kontrolle	0.2697	0.4495	0.3082	0.3835
skewness_R57	Pae vs Kontrolle	1.7044	6.2837	0.0000	0.0000
skewness_R58	Sepi vs Pae	-1.5044	-2.3139	0.0000	0.0000
skewness_R58	Sepi vs Kontrolle	0.3036	0.4979	0.2520	0.3240
skewness_R58	Pae vs Kontrolle	1.8080	6.4718	0.0000	0.0000
skewness_R60	Sepi vs Pae	-1.4672	-2.2109	0.0006	0.0010
skewness_R60	Sepi vs Kontrolle	0.2877	0.4817	0.2820	0.3541
skewness_R60	Pae vs Kontrolle	1.7549	6.2302	0.0000	0.0000
skewness_R62	Sepi vs Pae	-1.5627	-2.3809	0.0002	0.0003
skewness_R62	Sepi vs Kontrolle	0.5180	0.8760	0.0222	0.0306
skewness_R62	Pae vs Kontrolle	2.0808	6.5220	0.0000	0.0000
diff_R33	Sepi vs Pae	-75064.2509	-1.2673	0.0076	0.0110
diff_R33	Sepi vs Kontrolle	96777.2900	2.0420	0.0002	0.0003
diff_R33	Pae vs Kontrolle	171841.5409	3.4638	0.0000	0.0000

diff_R34	Sepi vs Pae	-75834.0127	-1.3380	0.0048	0.0070
diff_R34	Sepi vs Kontrolle	86573.0017	2.1249	0.0002	0.0003
diff_R34	Pae vs Kontrolle	162407.0144	3.4328	0.0000	0.0000
rel_diff_R33	Sepi vs Pae	-0.0730	-1.1308	0.0130	0.0184
rel_diff_R33	Sepi vs Kontrolle	0.1061	1.8970	0.0002	0.0003
rel_diff_R33	Pae vs Kontrolle	0.1791	3.4191	0.0000	0.0000
rel_diff_R34	Sepi vs Pae	-0.0756	-1.2491	0.0104	0.0148
rel_diff_R34	Sepi vs Kontrolle	0.0939	1.9936	0.0000	0.0000
rel_diff_R34	Pae vs Kontrolle	0.1696	3.4328	0.0000	0.0000
frac_diff_R3	Sepi vs Pae	-0.1113	-2.2844	0.0000	0.0000
frac_diff_R3	Sepi vs Kontrolle	0.0618	1.1575	0.0148	0.0208
frac_diff_R3	Pae vs Kontrolle	0.1731	4.5260	0.0000	0.0000
frac_diff_R33	Sepi vs Pae	-0.1382	-2.8961	0.0000	0.0000
frac_diff_R33	Sepi vs Kontrolle	0.0876	1.8934	0.0000	0.0000
frac_diff_R33	Pae vs Kontrolle	0.2258	7.7549	0.0000	0.0000
frac_diff_R34	Sepi vs Pae	-0.1255	-2.5685	0.0000	0.0000
frac_diff_R34	Sepi vs Kontrolle	0.0860	1.8362	0.0000	0.0000
frac_diff_R34	Pae vs Kontrolle	0.2115	7.9159	0.0000	0.0000
frac_diff_R45	Sepi vs Pae	-0.0842	-1.5731	0.0016	0.0024
frac_diff_R45	Sepi vs Kontrolle	0.0838	1.5417	0.0018	0.0027
frac_diff_R45	Pae vs Kontrolle	0.1680	3.6102	0.0000	0.0000
log_diff_R33	Sepi vs Pae	-0.0624	-1.1350	0.0170	0.0238
log_diff_R33	Sepi vs Kontrolle	0.0968	1.9142	0.0002	0.0003
log_diff_R33	Pae vs Kontrolle	0.1592	3.4716	0.0000	0.0000
log_diff_R34	Sepi vs Pae	-0.0645	-1.2519	0.0098	0.0141
log_diff_R34	Sepi vs Kontrolle	0.0858	2.0154	0.0000	0.0000
log_diff_R34	Pae vs Kontrolle	0.1503	3.5213	0.0000	0.0000
sample_startslope_R2	Sepi vs Pae	-11533.0603	-5.1498	0.0000	0.0000
sample_startslope_R2	Sepi vs Kontrolle	-104.0792	-0.0705	0.8670	0.9326
sample_startslope_R2	Pae vs Kontrolle	11428.9811	5.6332	0.0000	0.0000
sample_startslope_R3	Sepi vs Pae	-8733.5833	-4.9007	0.0000	0.0000
sample_startslope_R3	Sepi vs Kontrolle	-24.4266	-0.0181	0.9686	0.9854
sample_startslope_R3	Pae vs Kontrolle	8709.1567	6.2009	0.0000	0.0000
sample_startslope_R4	Sepi vs Pae	-8633.5092	-4.5138	0.0000	0.0000
sample_startslope_R4	Sepi vs Kontrolle	-157.4753	-0.1162	0.7878	0.8790
sample_startslope_R4	Pae vs Kontrolle	8476.0338	5.5408	0.0000	0.0000
sample_startslope_R17	Sepi vs Pae	-57392.5991	-4.6050	0.0000	0.0000
sample_startslope_R17	Sepi vs Kontrolle	660.1329	0.0879	0.8280	0.9051
sample_startslope_R17	Pae vs Kontrolle	58052.7321	5.3348	0.0000	0.0000
sample_startslope_R18	Sepi vs Pae	-56941.3931	-4.7755	0.0000	0.0000
sample_startslope_R18	Sepi vs Kontrolle	1743.5455	0.2230	0.5864	0.6973
sample_startslope_R18	Pae vs Kontrolle	58684.9386	5.5361	0.0000	0.0000
sample_startslope_R21	Sepi vs Pae	-9344.8849	-4.9313	0.0000	0.0000
sample_startslope_R21	Sepi vs Kontrolle	260.4112	0.1671	0.7136	0.8199
sample_startslope_R21	Pae vs Kontrolle	9605.2961	6.3853	0.0000	0.0000
sample_startslope_R24	Sepi vs Pae	-13269.2046	-4.6037	0.0000	0.0000
sample_startslope_R24	Sepi vs Kontrolle	13.7552	0.0084	0.9832	0.9854
sample_startslope_R24	Pae vs Kontrolle	13282.9598	5.2381	0.0000	0.0000
sample_startslope_R29	Sepi vs Pae	-10633.8360	-5.2784	0.0000	0.0000

sample_startslope_R29	Sepi vs Kontrolle	-251.8155	-0.1746	0.6756	0.7829
sample_startslope_R29	Pae vs Kontrolle	10382.0205	5.9889	0.0000	0.0000
sample_startslope_R30	Sepi vs Pae	-10670.8306	-5.0920	0.0000	0.0000
sample_startslope_R30	Sepi vs Kontrolle	23.2329	0.0154	0.9706	0.9854
sample_startslope_R30	Pae vs Kontrolle	10694.0635	6.1830	0.0000	0.0000
sample_startslope_R31	Sepi vs Pae	-8854.2312	-4.8778	0.0000	0.0000
sample_startslope_R31	Sepi vs Kontrolle	-70.3211	-0.0689	0.8794	0.9365
sample_startslope_R31	Pae vs Kontrolle	8783.9101	5.5238	0.0000	0.0000
sample_startslope_R35	Sepi vs Pae	-29923.2254	-5.0170	0.0000	0.0000
sample_startslope_R35	Sepi vs Kontrolle	-273.9155	-0.0754	0.8628	0.9318
sample_startslope_R35	Pae vs Kontrolle	29649.3099	5.7860	0.0000	0.0000
sample_startslope_R36	Sepi vs Pae	-28724.0272	-5.0615	0.0000	0.0000
sample_startslope_R36	Sepi vs Kontrolle	-149.1320	-0.0414	0.9236	0.9666
sample_startslope_R36	Pae vs Kontrolle	28574.8953	5.9237	0.0000	0.0000
sample_startslope_R37	Sepi vs Pae	-39242.8320	-5.0408	0.0000	0.0000
sample_startslope_R37	Sepi vs Kontrolle	254.2767	0.0523	0.9054	0.9549
sample_startslope_R37	Pae vs Kontrolle	39497.1087	5.8605	0.0000	0.0000
sample_startslope_R38	Sepi vs Pae	-40389.0223	-5.0831	0.0000	0.0000
sample_startslope_R38	Sepi vs Kontrolle	-312.2973	-0.0677	0.8738	0.9362
sample_startslope_R38	Pae vs Kontrolle	40076.7250	5.6277	0.0000	0.0000
sample_startslope_R39	Sepi vs Pae	-49868.3670	-4.7626	0.0000	0.0000
sample_startslope_R39	Sepi vs Kontrolle	-838.0368	-0.1372	0.7500	0.8544
sample_startslope_R39	Pae vs Kontrolle	49030.3302	5.3446	0.0000	0.0000
sample_startslope_R40	Sepi vs Pae	-48944.6841	-4.9195	0.0000	0.0000
sample_startslope_R40	Sepi vs Kontrolle	-100.4654	-0.0179	0.9676	0.9854
sample_startslope_R40	Pae vs Kontrolle	48844.2187	5.5455	0.0000	0.0000
sample_startslope_R41	Sepi vs Pae	-51812.1659	-4.7961	0.0000	0.0000
sample_startslope_R41	Sepi vs Kontrolle	-92.8580	-0.0149	0.9716	0.9854
sample_startslope_R41	Pae vs Kontrolle	51719.3079	5.1964	0.0000	0.0000
sample_startslope_R42	Sepi vs Pae	-51962.3453	-4.8805	0.0000	0.0000
sample_startslope_R42	Sepi vs Kontrolle	-155.2798	-0.0254	0.9526	0.9854
sample_startslope_R42	Pae vs Kontrolle	51807.0655	5.1797	0.0000	0.0000
sample_startslope_R43	Sepi vs Pae	-52295.4096	-5.1081	0.0002	0.0003
sample_startslope_R43	Sepi vs Kontrolle	-69.3650	-0.0111	0.9816	0.9854
sample_startslope_R43	Pae vs Kontrolle	52226.0447	5.9071	0.0000	0.0000
sample_startslope_R44	Sepi vs Pae	-54068.2714	-5.1456	0.0000	0.0000
sample_startslope_R44	Sepi vs Kontrolle	-689.0681	-0.1176	0.7856	0.8790
sample_startslope_R44	Pae vs Kontrolle	53379.2033	5.7514	0.0000	0.0000
sample_startslope_R45	Sepi vs Pae	-48446.4067	-5.1501	0.0000	0.0000
sample_startslope_R45	Sepi vs Kontrolle	-766.8415	-0.1516	0.7370	0.8432
sample_startslope_R45	Pae vs Kontrolle	47679.5653	5.8282	0.0000	0.0000
sample_startslope_R46	Sepi vs Pae	-53511.8535	-5.0504	0.0000	0.0000
sample_startslope_R46	Sepi vs Kontrolle	44.8635	0.0076	0.9854	0.9854
sample_startslope_R46	Pae vs Kontrolle	53556.7170	5.7494	0.0000	0.0000
sample_startslope_R47	Sepi vs Pae	-58981.3264	-4.8274	0.0000	0.0000
sample_startslope_R47	Sepi vs Kontrolle	-383.9100	-0.0556	0.8968	0.9496
sample_startslope_R47	Pae vs Kontrolle	58597.4164	5.4060	0.0000	0.0000
sample_startslope_R50	Sepi vs Pae	-28320.5732	-5.1708	0.0000	0.0000
sample_startslope_R50	Sepi vs Kontrolle	-74.3462	-0.0202	0.9646	0.9854

sample_startslope_R50	Pae vs Kontrolle	28246.2270	6.1764	0.0000	0.0000
sample_startslope_R51	Sepi vs Pae	-41292.0253	-5.1877	0.0000	0.0000
sample_startslope_R51	Sepi vs Kontrolle	-561.4777	-0.1225	0.7830	0.8790
sample_startslope_R51	Pae vs Kontrolle	40730.5476	5.7032	0.0000	0.0000
sample_startslope_R52	Sepi vs Pae	-40981.7873	-5.2696	0.0000	0.0000
sample_startslope_R52	Sepi vs Kontrolle	-516.5242	-0.1086	0.8010	0.8864
sample_startslope_R52	Pae vs Kontrolle	40465.2630	5.9277	0.0000	0.0000
sample_startslope_R53	Sepi vs Pae	-50122.3610	-4.9521	0.0000	0.0000
sample_startslope_R53	Sepi vs Kontrolle	-481.1641	-0.0769	0.8536	0.9256
sample_startslope_R53	Pae vs Kontrolle	49641.1969	5.2968	0.0000	0.0000
sample_startslope_R54	Sepi vs Pae	-48637.9382	-4.8669	0.0000	0.0000
sample_startslope_R54	Sepi vs Kontrolle	228.7019	0.0374	0.9276	0.9670
sample_startslope_R54	Pae vs Kontrolle	48866.6401	5.5015	0.0000	0.0000
sample_startslope_R56	Sepi vs Pae	-54230.3166	-5.1426	0.0000	0.0000
sample_startslope_R56	Sepi vs Kontrolle	-531.2560	-0.0936	0.8336	0.9075
sample_startslope_R56	Pae vs Kontrolle	53699.0605	5.7561	0.0000	0.0000
sample_startslope_R57	Sepi vs Pae	-53175.3789	-5.0332	0.0000	0.0000
sample_startslope_R57	Sepi vs Kontrolle	-1099.2646	-0.1938	0.6512	0.7579
sample_startslope_R57	Pae vs Kontrolle	52076.1143	5.3789	0.0000	0.0000
sample_startslope_R58	Sepi vs Pae	-54140.0581	-4.9808	0.0000	0.0000
sample_startslope_R58	Sepi vs Kontrolle	-1060.6189	-0.1861	0.6440	0.7527
sample_startslope_R58	Pae vs Kontrolle	53079.4391	5.3887	0.0000	0.0000
sample_startslope_R59	Sepi vs Pae	-51869.1487	-5.1307	0.0000	0.0000
sample_startslope_R59	Sepi vs Kontrolle	361.5767	0.0623	0.8810	0.9365
sample_startslope_R59	Pae vs Kontrolle	52230.7254	5.9081	0.0000	0.0000
sample_startslope_R60	Sepi vs Pae	-52583.8715	-4.9757	0.0000	0.0000
sample_startslope_R60	Sepi vs Kontrolle	-275.5344	-0.0447	0.9168	0.9632
sample_startslope_R60	Pae vs Kontrolle	52308.3371	5.5551	0.0000	0.0000
sample_startslope_R61	Sepi vs Pae	-46009.0051	-4.9223	0.0000	0.0000
sample_startslope_R61	Sepi vs Kontrolle	-1481.3633	-0.2228	0.5934	0.6996
sample_startslope_R61	Pae vs Kontrolle	44527.6418	5.2422	0.0000	0.0000
recov_slope_R33	Sepi vs Pae	1712.9312	1.4219	0.0020	0.0030
recov_slope_R33	Sepi vs Kontrolle	-1672.4608	-1.8800	0.0000	0.0000
recov_slope_R33	Pae vs Kontrolle	-3385.3920	-3.4968	0.0000	0.0000
recov_slope_R34	Sepi vs Pae	1820.7744	2.0344	0.0000	0.0000
recov_slope_R34	Sepi vs Kontrolle	-1385.7698	-1.9443	0.0000	0.0000
recov_slope_R34	Pae vs Kontrolle	-3206.5441	-4.1412	0.0000	0.0000
sample_endslope_R55	Sepi vs Pae	244.3815	0.1207	0.8104	0.8931
sample_endslope_R55	Sepi vs Kontrolle	-2028.6372	-1.1743	0.0004	0.0006
sample_endslope_R55	Pae vs Kontrolle	-2273.0187	-1.8009	0.0000	0.0000

Table S6: Results of the permutation tests. Every feature in this table was test for significant differences between the classes listes in the column “Comparison”.
